# The *R^r^* Form of the Kedem–Katchalsky–Peusner Model Equations for Description of the Membrane Transport in Concentration Polarization Conditions

**DOI:** 10.3390/e22080857

**Published:** 2020-08-01

**Authors:** Kornelia M. Batko, Andrzej Ślęzak, Sławomir Grzegorczyn, Wioletta M. Bajdur

**Affiliations:** 1 Department of Business Informatics, University of Economics, 40287 Katowice, Poland; 2 Department of Innovation and Safety Management Systems, Technical University of Czestochowa, 42200 Czestochowa, Poland; aslezak52@gmail.com; 3 Department of Biophysics, Faculty of Medicine with the Division of Dentistry in Zabrze, Medical University of Silesia, 19 H. Jordan Str., 41808 Zabrze, Poland; grzegorczyn@sum.edu.pl

**Keywords:** membrane transport, non-electrolyte solutions, Peusner’s network thermodynamics, Kedem–Katchalsky equations, concentration polarization

## Abstract

The paper presents the $R^{r}$ matrix form of Kedem–Katchalsky–Peusner equations for membrane transport of the non-homogeneous ternary non-electrolyte solutions. Peusner’s coefficients $R_{ij}^{r}$ and *det* [$R^{r}$] (*i*, *j* ∈ {1, 2, 3}, *r* = A, B) occurring in these equations, were calculated for Nephrophan biomembrane, glucose in aqueous ethanol solutions and two different settings of the solutions relative to the horizontally oriented membrane for concentration polarization conditions or homogeneity of solutions. Kedem–Katchalsky coefficients, measured for homogeneous and non-homogeneous solutions, were used for the calculations. The calculated Peusner’s coefficients for homogeneous solutions depend linearly, and for non-homogeneous solutions non-linearly on the concentrations of solutes. The concentration dependences of the coefficients $R_{ij}^{r}$ and *det* [$R^{r}$] indicate a characteristic glucose concentration of 9.24 mol/m^3^ (at a fixed ethanol concentration) in which the obtained curves for Configurations A and B intersect. At this point, the density of solutions in the upper and lower membrane chamber are the same. Peusner’s coefficients were used to assess the effect of concentration polarization and free convection on membrane transport (the *ξ_ij_* coefficient), determine the degree of coupling (the $r_{ij}^{r}$ coefficient) and coupling parameter (the $Q_{R}^{r}$ coefficient) and energy conversion efficiency (the ${\left(e_{ij}^{r}\right)}_{r}$ coefficient).

## 1. Introduction

Membrane transport belongs to the group of processes described by thermodynamics of irreversible processes, now called modern thermodynamics. This theory was created and described by Lars Onsager, Theophile De Donder, Ilya Prigogine and others [1]. This field of knowledge has provided many research tools for transport mechanisms, including membrane transport, which is used in many areas of science (physics, biology, chemistry) and technology (biotechnology, biomedical engineering, water and sewage engineering, bioenergetics) [2,3,4,5,6,7,8,9]. One of the basic research tools for membrane transport are the Kedem–Katchalsky Equations (K–K Equations) derived from Onsager thermodynamics. The K–K Equations show the relationship between volume (*J_v_*), solute (*J_s_*) fluxes and thermodynamic forces (osmotic Δ*π* and/or hydrostatic Δ*P*) [10,11]. Currently, several versions of these equations classical form [12] and forms presented by Kargol and Kargol [13,14], Peusner [15], Elmoazzen et al. [16], Cheng and Pinsky [17] and Cardoso and Cartwright [18].

The starting point of Onsager thermodynamics is the scattering function: Φ = *T*(*d_i_S*/*dt*), where *T* is the absolute temperature, and *d_i_S*/*dt* − the production of internal entropy [8,11]. For isothermal processes: $\Phi =\underset{i}{\sum }J_{i}X_{i}$. If the forces (*X_i_*) and flows (*J_i_*) are related by linear equations in the form $X_{i}=\underset{j}{\sum }R_{ij}J_{j}$, then the matrix of coefficients *R* is symmetrical, i.e., *R_ij_* = *R_ji_*. The degree of coupling *r_ij_* results from the relationship between forces and fluxes [19,20] and for diluted and homogeneous solutions is determined by the relations *r_ij_* = –*R_ij_*(*R_ii_R_jj_*)^−0.5^ and *r_ij_* = *r_ji_* = *r*. The second law of thermodynamics imposes the condition *R_ii_R_jj_* ≥ (*R_ij_*)^2^, which means that *r_ij_* is limited by the relation −1 ≤ *r* ≤ +1. When *r* = ± 1, the system is completely coupled, the processes become a single process. When *r* = 0, the two processes are completely unrelated and there are no energy conversion interactions. Considering the *r* factor, Kedem and Caplan presented the expression of the maximum energy conversion efficiency: *e_max_* = *r*^2^[1 + (1 – *r*^2^)^0.5^]^−2^ [16]. In turn, Peusner proposed a coupling parameter called “super *Q_R_*”: *Q_R_* = *r*^2^(2 – *r*^2^)^−1^ [15,21].

The network form of K–K Equations was presented by Leonardo Peusner [21,22]. He obtained these equations as a result of symmetrical and/or hybrid transformation of classic Kedem–Katchalsky equations with the use of network thermodynamics, which he developed (Peusner NT) [23]. It should be noted that network thermodynamics developed by Oster, Perelson and Katchalsky (Oster, Perelson, Katchalsky NT) also occurs in science [24]. For homogeneous and non-homogeneous binary solutions of nonelectrolytes, there are two symmetrical and two hybrid forms of K–K Equations. Symmetrical forms of these equations contain Peusner matrix coefficients: *R_ij_* and *L_ij_* (for homogeneous solutions) and $R_{ij}^{r}$ and $L_{ij}^{r}$ (for non-homogeneous solutions), while hybrid forms include Peusner coefficients: *P_ij_* and *H_ij_* (for homogeneous solutions) and $P_{ij}^{r}$ and $H_{ij}^{r}$ (for non-homogeneous solutions) (*i*, *j* ∈ {1, 2}) [25,26,27,28]. It should be noted that solutions which are vigorously mechanically stirred are considered as homogeneous solutions [29,30]. In turn, for heterogeneous solutions (solutions in which concentration polarization occurs), consisting in the formation of concentration boundary layers (CBLs) on both sides of the membrane separating solutions [31,32,33,34,35,36,37,38]. These layers serve as additional kinetic barriers for rapidly penetrating substances through membranes in artificial and biological systems [37,38,39,40,41]. For multicomponent solutions, the number of Peusner matrix coefficients increases: for ternary solutions, there are eight Peusner coefficients: *R_ij_*, *L_ij_*, *H_ij_*, *N_ij_*, *K_ij_*, *P_ij_*, *S_ij_* and *W_ij_*—for homogeneous solutions and $R_{ij}^{r}$, $L_{ij}^{r}$, $H_{ij}^{r}$, $N_{ij}^{r}$, $K_{ij}^{r}$, $P_{ij}^{r}$, $S_{ij}^{r}$ or $W_{ij}^{r}$—for nonhomogeneous solutions (*i*, *j* ∈ {1, 2, 3}, *r* = A or B) [42]. It should be noted that the symmetrical forms of these K–K Equations, as in the case of binary solutions, include Peusner coefficients $R_{ij}^{r}$ or $L_{ij}^{r}$, while hybrid forms—other Peusner coefficients. It should be noted that the coefficients $R_{ij}^{r}$ or $L_{ij}^{r}$, come directly from Onsager thermodynamics, and the remaining coefficients are a consequence of the application of network thermodynamics techniques [25,26,27,28,42,43].

In the previous papers [42,43] the case of two directional port of Peusner’s network thermodynamics with single inputs for volume flux $J_{v}^{r}$ coupled with thermodynamic force $\Delta P-\Delta \pi_{1}-\Delta \pi_{2}$ and solute fluxes: $J_{1}^{r}$ coupled with thermodynamic force $\Delta \pi_{1}/{\bar{C}}_{1}$ and $J_{2}^{r}$ coupled with thermodynamic force $\Delta \pi_{2}/{\bar{C}}_{2}$ was considered. The network K–K Equations for non-homogeneous ternary non-electrolyte solutions containing Peusner’s coefficients $H_{ij}^{r}$ and $L_{ij}^{r}$ (*i*, *j* ∈ {1, 2, 3}, *r* = A, B) were obtained by means of hybrid network transformations of Peusner’s network thermodynamic. The coefficients $H_{ij}^{r}$ and $L_{ij}^{r}$ (*i*, *j* ∈ {1, 2, 3}, *r* = A, B) occurring in the matrix [$H^{r}$] and [$L^{r}$] we call Peusner’s coefficients and matrix [$H^{r}$] or [$L^{r}$]—matrix of Peusner’s coefficients $H_{ij}^{r}$ or $L_{ij}^{r}$ respectively. According to the principles of network thermodynamic, for non-diagonal coefficients we have $H_{12}^{r}$ ≠ $H_{21}^{r}$, $H_{13}^{r}$ ≠ $H_{31}^{r}$, $H_{23}^{r}$ ≠ $H_{32}^{r}$, $L_{12}^{r}$ ≠ $L_{21}^{r}$, $L_{13}^{r}$ ≠ $L_{31}^{r}$ and $L_{23}^{r}$ ≠ $L_{32}^{r}$.

The aim of this paper is to develop the form of $R^{r}$ of the K–K Equations, containing the Peusner coefficients $R_{ij}^{r}$ (*i*, *j* ∈ {1, 2, 3}, *r* = A, B). We will present the results of calculations of coefficients $R_{ij}^{r}$ and $R_{ij}$ matrix coefficients $R_{det}^{r}$ = *det* [$R^{r}$] and $R_{det}$ = *det* [R] and the quotients ξ*_ij_* = ($R_{ij}^{A}$ − $R_{ij}^{B}$)/$R_{ij}$ and $\xi_{det}$ = ($R_{det}^{A}$ − $R_{det}^{B}$)/$R_{det}$ which were obtained on the basis of experimentally determined coefficients (*L_p_*, *σ*_1_, *σ*_2_, *ω*_11_, *ω*_22_, *ω*_21_, *ω*_12_, $\zeta_{1}^{r}$ and $\zeta_{2}^{r}\;)$ for glucose in aqueous ethanol solutions and Configurations A and B of the membrane system. These coefficients were calculated on the basis of experimentally measured volume ($J_{v}^{r}$) and solute fluxes ($J_{k}^{r}$) (*k* = 1, 2 and *r* = A, B) using the procedure described in [11,30,34]. Besides, we will present the results of calculations of the degree of coupling $r_{ij}=-R_{ij}{\left(R_{ii}R_{jj}\right)}^{-0.5}$ (for homogeneous ternary nonelectrolyte solutions), $r_{ij}^{r}=R_{ij}^{r}{\left(R_{ii}^{r}R_{jj}^{r}\right)}^{-0.5}$ (for non-homogeneous ternary nonelectrolyte solutions), coupling parameter $Q_{R}=r_{ij}r_{ji}{\left(2-r_{ij}r_{ji}\right)}^{-1}$ (for homogeneous ternary nonelectrolyte solutions), $Q_{R}^{r}=r_{ij}^{r}r_{ji}^{r}{\left(2-r_{ij}^{r}r_{ji}^{r}\right)}^{-1}$ (for non-homogeneous ternary nonelectrolyte solutions) and energy conversion coefficients ${\left(e_{ij}\right)}_{r}={\left(r_{ji}\right)}^{2}{\left[1+{\left(1-r_{ij}r_{ji}\right)}^{0.5}\right]}^{-2}$ (for homogeneous ternary nonelectrolyte solutions) and ${\left(e_{ij}^{r}\right)}_{r}={\left(r_{ji}^{r}\right)}^{2}{\left[1+{\left(1-r_{ij}^{r}r_{ji}^{r}\right)}^{0.5}\right]}^{-2}$ (for non-homogeneous ternary nonelectrolyte solutions) in which (*i*, *j* ∈ {1, 2, 3}, *r* = A, B).

## 2. Theory

**Figure 1:**
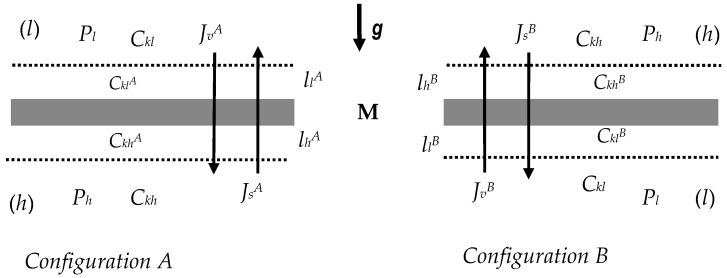
The model of single-membrane system: M—membrane, ***g***—gravitational acceleration,$\;l_{l}^{A}$ and $l_{h}^{A}$ —the concentration boundary layers in Configuration A, $l_{l}^{B}$ and $l_{h}^{B}$ —the concentration boundary layers in Configuration B, *P_h_* and *P_l_*—mechanical pressures, *C_kh_* and *C_kl_*—global solution concentrations, $C_{kl}^{A}$, $C_{kh}^{A}$, $C_{kl}^{B}$ and $\;C_{kh}^{B}$ —local (at boundaries between membrane and CBLs) solution concentrations,$\;J_{k}^{A}$ and $J_{v}^{A}$ —solute and volume fluxes in Configuration A, $J_{k}^{B}$ and $J_{v}^{B}$ —solute and volume fluxes in Configuration B.

Similarly, as in previous papers (e.g., [42,43]), let us consider the membrane system presented in Figure 1. In this system the membrane (M) is located in horizontal plane and separates compartments (*l*) and (*h*) filled with non-homogeneous ternary non-electrolyte solutions with concentrations at the initial moment (*t* = 0) *C_kh_* and *C_kl_* (*C_kh_* > *C_kl_*, *k* = 1, 2). This membrane treated as a “black box” type is isotropic, symmetrical, electroneutral and selective for solvent and non-ionized dissolved substances. For a membrane located in a horizontal plane that is perpendicular to the gravity vector, two configurations of the membrane system are possible. These configurations are denoted by A and B. In Configuration A, the *C_kl_* solution is in the chamber above the membrane, and the *C_kh_* solution is in the chamber under the membrane. In Configuration B, the arrangement of the solutions relative to the membrane is reversed.

We will consider only isothermal and stationary processes of membrane transport, for which the measure is the volume fluxes ($J_{v}^{r}$) and solutes fluxes ($J_{k}^{r}$) (*k* = 1, 2 and *r* = A, B). These fluxes can be described by the K–K Equations for ternary non-electrolyte solutions [42,43]. Under such conditions water and solutes which diffuse through the membrane create concentration boundary layers (CBLs), $l_{h}^{r}$ and $l_{l}^{r}$ on both sides of the membrane [35,36,37]. The thicknesses of $l_{h}^{r}$ and $l_{l}^{r}$ are equal suitably to $\delta_{h}^{r}$ and $\delta_{l}^{r}$. The mean concentrations of solutes „1” and „2” in membrane (${\bar{C}}_{1}$, $\;{\bar{C}}_{2}$) can be calculated using expressions ${\bar{C}}_{k}$ = (*C_kh_* – *C_kl_*)[ln(*C_kh_C_kl_*^−1^)]^−1^ (*k* = 1, 2). Appearance of CBLs causes that concentrations at the interfaces of the membrane and solutions respectively decreases from *C_kh_* to $C_{kh}^{r}$ and increases from *C_kl_* to $C_{kl}^{r}$ ($C_{kh}^{r}$ > $C_{kl}^{r}\;$, $C_{kl}^{r}\;$ > *C_kl_*, *C_kh_* > $C_{kh}^{r}$. *k* = 1, 2).

Let us denote by $\rho_{l}^{r}$ and $\rho_{h}^{r}$ the densities of solutions in the interfaces $l_{l}^{r}$/M and M/$l_{h}^{r}$ while by *ρ_l_* and *ρ_h_* (*ρ_l_* < *ρ_h_* or *ρ_l_* > *ρ_h_*) the density of solutions outside the CBLs. The following conditions can be saved for these densities: $\rho_{l}^{r}$ > *ρ_l_* or $\rho_{l}^{r}$ < $\rho_{h}^{r}$, $\rho_{l}^{r}$ > $\rho_{h}^{r}$ or $\rho_{l}^{r}$ < $\rho_{h}^{r}$ and $\rho_{h}^{r}\;$ > *ρ_h_* or $\rho_{h}^{r}$ < *ρ_h_*. If the solution with lower density is under the membrane, the system $l_{h}^{r}$/M/$l_{l}^{r}$ loses its hydrodynamic stability and convective instabilities in near membrane area are observed [35,36,37]. The measure of the concentration polarization (CP) is the CP coefficient ($\zeta_{k}^{r}$). Using this coefficient, we can write the relation: $C_{kh}^{r}-C_{kl}^{r}\;$ = $\zeta_{k}^{r}$ (*C_kh_* − *C_kl_*). The value of coefficient $\zeta_{k}^{r}$ depends on both the concentration of solutions separated by the membrane $\left({\bar{C}}_{k}\right)$ and the configuration of the membrane system (*r* = A, B). More specifically for this case, the thicknesses of CBLs $\delta_{h}^{r}$ and $\delta_{l}^{r}$ exceed values ($\delta_{h}^{r}$)_crit_ and ($\delta_{l}^{r}$)_crit_ and CP coefficient ($\zeta_{k}^{r}$) exceed its critical value ($\zeta_{k}^{r})\;$_crit_ suitably [42,43]. The dependency between the CP coefficient ($\zeta_{k}^{r}$) and the thickness of CBLs ($\delta_{h}^{r}$ and $\delta_{l}^{r}$) can be described by the following expression [37].

(1) $$\zeta_{k}^{r}={\left\{1+RT\omega_{ij}\left[\frac{\delta_{l}^{r}}{{\left(D_{ij}^{r}\right)}_{l}}+\frac{\delta_{h}^{r}}{{\left(D_{ij}^{r}\right)}_{h}}\right]\right\}}^{-1}$$

where (*i*, *j* ∈ {1, 2} and *r* = A, B). In diluted non-electrolyte solutions, the diffusion coefficients ${\left(D_{ks}^{r}\right)}_{l}$ and ${\left(D_{ks}^{r}\right)}_{h}$ are independent both of gravitational direction and solution concentration. Therefore, we can assume that ${\left(D_{ks}^{r}\right)}_{l}$ = ${\left(D_{ks}^{r}\right)}_{h}$ = *D_ks_*. Besides, we can also assume that $\delta_{h}^{r}$ = $\delta_{l}^{r}$ = *δ^r^*.

According to the Kedem–Katchalsky formalism [11] transport properties of the membrane are determined for solutions containing a solvent and two dissolved substances (ternary solution) by practical coefficients: hydraulic permeability (*L_p_*), reflection (*σ_k_*, *k* = 1, 2) and permeability of solute (*ω_kf_*, *k*, *f* ∈ {1, 2}). In turn, the transport properties of the complex $l_{h}^{r}$/M/$l_{l}^{r}$ are characterized by coefficients of hydraulic permeability ($L_{p}^{r}$), reflection ($\sigma_{sk}^{r}$, $\sigma_{ak}^{r}$) and permeability of solute ($\omega_{kf}^{r}$). The coefficients of hydraulic, osmotic, advective and diffusive concentration polarization are defined by expressions: $\zeta_{p}^{r}$ = $L_{p}^{r}$/*L_p_*, $\zeta_{v}^{r}$ = $\sigma_{sk}^{r}$/*σ_k_*, $\zeta_{a}^{r}$ = $\sigma_{ak}^{r}$/*σ_k_* and $\zeta_{k}^{r}$ = $\omega_{kf}^{r}$/*ω_kf_* [26]. For osmotic volume and diffusive fluxes of homogeneous (evenly stirred) solutions, the values of volume (*J_v_*) and solute (*J_k_*) fluxes does not depend on the configuration of the membrane system. Besides, the dependencies *J_v_* = *f*(*C_kh_* − *C_kl_*) and *J_k_* = *f*(*C_kh_ − C_kl_*) are linear, while $J_{v}^{r}$ = *f*(*C_kh_ − C_kl_*) and $J_{k}^{r}$ = *f*(*C_kh_ − C_kl_*) are nonlinear [33,43]. The formation of the layers $l_{l}^{r}$ and $l_{h}^{r}$ reduce the value of volume and solute fluxes from *J_v_* and *J_k_* (in conditions of homogeneous solutions) to $J_{v}^{r}$ and $J_{k}^{r}$ (in condition of CP), respectively.

The Kedem–Katchalsky Equations for CP conditions can be written as:

(2) $$J_{v}^{r}=\zeta_{p}^{r}L_{p}\left(\Delta P-\zeta_{v1}^{r}\sigma_{1}\Delta \pi_{1}-\zeta_{v2}^{r}\sigma_{2}\Delta \pi_{2}\right)$$

(3) $$J_{1}^{r}=\zeta_{s11}^{r}\omega_{11}\Delta \pi_{1}+\zeta_{s12}^{r}\omega_{12}\Delta \pi_{2}+{\bar{C}}_{1}\left(1-\zeta_{a1}^{r}\sigma_{1}\right)J_{v}^{r}$$

(4) $$J_{2}^{r}=\zeta_{s21}^{r}\omega_{21}\Delta \pi_{1}+\zeta_{s22}^{r}\omega_{22}\Delta \pi_{2}+{\bar{C}}_{1}\left(1-\zeta_{a2}^{r}\sigma_{2}\right)J_{v}^{r}$$

where $J_{v}^{r}$, $J_{1}^{r}$ and $J_{2}^{r}$—volume and solutes „1” and „2” fluxes respectively, *L_p_*—hydraulic permeability coefficient, *σ*_1_ and *σ*_2_—reflection coefficients suitably for solutes „1” or „2”, *ω*_11_ and *ω*_22_—solute permeability coefficients for solutes „1” or „2” generated by forces with indexes „1” or „2” and *ω*_12_ and *ω*_21_—cross coefficients of permeability for substances „1” or „2” generated by forces with indexes „2” or „1” respectively. Δ*P* = *P_h_* − *P_l_* is the hydrostatic pressure difference (*P_h_*, *P_l_* are higher and lower values of hydrostatic pressure suitably). Δ*π_k_* = *RT* (*C_kh_* − *C_kl_*) is the difference of osmotic pressure (*RT* is the product of gas constant and thermodynamic temperature whereas *C_kh_* and *C_kl_* are solutes concentrations, *k* = 1, 2). ${\bar{C}}_{k}$ is the mean solute concentration in membrane and is expressed by ${\bar{C}}_{k}$ = (*C_kh_* − *C_kl_*)[ln(*C_kh_C_kl_*^−1^)]^−1^ (*k* = 1, 2). By means of this expression one can show that Δ*π_k_*/${\bar{C}}_{k}$ = ln (*C_kh_C_kl_*^−1^). Equations (2)–(4) are modified Kedem–Katchalsky Equations for ternary solutions [33].

The Equations (2)–(4) can be transformed by simple algebraic transformations to the matrix form of the Kedem–Katchalsky–Peusner equations for non-homogenous non-electrolyte ternary solutions:

(5) $$\left[\begin{array}{c} \Delta P-\Delta \pi_{1}-\Delta \pi_{2}\\ \frac{\Delta \pi_{1}}{{\bar{C}}_{1}}\\ \frac{\Delta \pi_{2}}{{\bar{C}}_{2}} \end{array}\right]=\left[\begin{array}{ccc} R_{11}^{r} & R_{12}^{r} & R_{13}^{r}\\ R_{21}^{r} & R_{22}^{r} & R_{23}^{r}\\ R_{31}^{r} & R_{32}^{r} & R_{33}^{r} \end{array}\right]\left[\begin{array}{c} J_{v}^{r}\\ J_{1}^{r}\\ J_{2}^{r} \end{array}\right]=\left[R^{r}\right]\left[\begin{array}{c} J_{v}^{r}\\ J_{1}^{r}\\ J_{2}^{r} \end{array}\right]$$

where $R_{11}^{r}={\left(\zeta_{p}^{r}L_{p}\right)}^{-1}-\left[\left(1-\zeta_{v1}^{r}\sigma_{1}\right)(\alpha_{1}-\alpha_{2})+\left(1-\zeta_{v2}^{r}\sigma_{2}\right)(\alpha_{3}-\alpha_{4})\right]\gamma^{-1}$, $\alpha_{1}=\zeta_{s12}^{r}\omega_{12}\left(1-\zeta_{a2}^{r}\sigma_{2}\right){\bar{C}}_{2}$, $\alpha_{2}=\zeta_{s22}^{r}\omega_{22}\left(1-\zeta_{a1}^{r}\sigma_{1}\right){\bar{C}}_{1}$, $\alpha_{3}=\zeta_{s21}^{r}\omega_{21}\left(1-\zeta_{a1}^{r}\sigma_{1}\right){\bar{C}}_{1}$, $\alpha_{4}=\zeta_{s11}^{r}\omega_{11}\left(1-\zeta_{a2}^{r}\sigma_{2}\right){\bar{C}}_{2}$, $\gamma =\zeta_{s11}^{r}\omega_{11}\zeta_{s22}^{r}\omega_{22}-\zeta_{s12}^{r}\omega_{12}\zeta_{s21}^{r}\omega_{21}$, $R_{12}^{r}=\left[\zeta_{s21}^{r}\omega_{21}\left(1-\zeta_{v2}^{r}\sigma_{2}\right)-\zeta_{s22}^{r}\omega_{22}\left(1-\zeta_{v1}^{r}\sigma_{1}\right)\right]\gamma^{-1}$, $R_{13}^{r}=\left[\zeta_{s12}^{r}\omega_{12}\left(1-\zeta_{v1}^{r}\sigma_{1}\right)-\zeta_{s11}^{r}\omega_{11}\left(1-\zeta_{v2}^{r}\sigma_{2}\right)\right]\gamma^{-1}$, $R_{21}^{r}=\left[\zeta_{s12}^{r}\omega_{12}\left(1-\zeta_{a2}^{r}\sigma_{2}\right){\bar{C}}_{2}-\zeta_{s22}^{r}\omega_{22}\left(1-\zeta_{a1}^{r}\sigma_{1}\right){\bar{C}}_{1}\right]\gamma^{-1}$, $R_{22}^{r}=\zeta_{s22}^{r}\omega_{22}\gamma^{-1}{\bar{C}}_{1}{}^{-1}$ $R_{23}^{r}=-\zeta_{s12}^{r}\omega_{12}\gamma^{-1}{\bar{C}}_{1}{}^{-1}$, $R_{31}^{r}=\left[\zeta_{s21}^{r}\left(1-\zeta_{a1}^{r}\sigma_{1}\right){\bar{C}}_{1}-\zeta_{s11}^{r}\omega_{11}\left(1-\zeta_{a2}^{r}\sigma_{2}\right){\bar{C}}_{2}\right]\gamma^{-1}$, $R_{32}^{r}=-\zeta_{s21}^{r}\omega_{21}\gamma^{-1}{\bar{C}}_{2}{}^{-1}$, $R_{33}^{r}=\zeta_{s11}^{r}\omega_{11}\gamma^{-1}{\bar{C}}_{2}{}^{-1}$, [*R^r^*] is the matrix of the Peusner’s coefficients $R_{ij}^{r}$ (i, j∈{1, 2, 3}) for ternary non-electrolyte solutions in conditions of concentration polarization.

Results from Equation (5) are the non-diagonal coefficients $R_{12}^{r}$ ≠ $R_{21}^{r}$, $R_{13}^{r}\;$ ≠ $R_{31}^{r}$ and $R_{23}^{r}$ ≠ $R_{32}^{r}$. Besides, the determinant of the matrix [*R^r^*] is equal to:

(6) $$det\;\left[R^{r}\right]=\frac{1}{\zeta_{p}^{r}L_{p}{\bar{C}}_{1}{\bar{C}}_{2}\left(\omega_{11}\zeta_{s11}^{r}\omega_{22}\zeta_{s22}^{r}-\omega_{12}\zeta_{s12}^{r}\omega_{21}\zeta_{s21}^{r}\right)}\equiv R_{det}^{r}$$

Index „*r*” in Equations (2)–(6) indicate that the fluxes $J_{v}^{r}$, $J_{1}^{r}$, $J_{2}^{r}$, Coefficients $R_{ij}^{r}$ (*i*, *j* ∈ {1, 2, 3} and matrix [*R^r^*] of these coefficients (*R^r^* form of the matrix of Peusner’s coefficients), depend on configuration of the membrane system (*r* = A, B). From a formal point of view, the case of $R_{det}^{r}$ = 0 is excluded, because in order for the denominator of Equation (6) to be different from zero, the condition $\omega_{11}\zeta_{s11}^{r}\omega_{22}\zeta_{s22}^{r}\neq \omega_{12}\zeta_{s12}^{r}\omega_{21}\zeta_{s21}^{r}$ must be satisfied. If $\omega_{11}\zeta_{s11}^{r}\omega_{22}\zeta_{s22}^{r}>\omega_{12}\zeta_{s12}^{r}\omega_{21}\zeta_{s21}^{r}$ then $R_{det}^{r}$ > 0, and if $\omega_{11}\zeta_{s11}^{r}\omega_{22}\zeta_{s22}^{r}<\omega_{12}\zeta_{s12}^{r}\omega_{21}\zeta_{s21}^{r}$ then $R_{det}^{r}$ < 0.

In order to write Equations (5) and (6) for the conditions of homogeneity of solutions, the superscript “r” should be removed and assumption that the condition $\zeta_{p}^{r}$ = $\zeta_{v1}^{r}$ = $\zeta_{v2}^{r}$ = $\zeta_{a1}^{r}$ = $\zeta_{a2}^{r}$ = $\zeta_{s11}^{r}$ = $\zeta_{s12}^{r}$ = $\zeta_{s22}^{r}$ = $\zeta_{s21}^{r}$ = 1 is fulfilled. Then Equations (5) and (6) are taking the following form:

(7) $$\left[\begin{array}{c} \Delta P-\Delta \pi_{1}-\Delta \pi_{2}\\ \frac{\Delta \pi_{1}}{{\bar{C}}_{1}}\\ \frac{\Delta \pi_{2}}{{\bar{C}}_{2}} \end{array}\right]=\left[\begin{array}{ccc} R_{11} & R_{12} & R_{13}\\ R_{21} & R_{22} & R_{23}\\ R_{31} & R_{32} & R_{33} \end{array}\right]\left[\begin{array}{c} J_{v}\\ J_{1}\\ J_{2} \end{array}\right]=\left[R\right]\left[\begin{array}{c} J_{v}\\ J_{1}\\ J_{2} \end{array}\right]$$

where $R_{11}^{r}={\left(\zeta_{p}^{r}L_{p}\right)}^{-1}-\left[\left(1-\zeta_{v1}^{r}\sigma_{1}\right)(\alpha_{1}-\alpha_{2})+\left(1-\zeta_{v2}^{r}\sigma_{2}\right)(\alpha_{3}-\alpha_{4})\right]\gamma^{-1}$, $\alpha_{1}=\omega_{12}\left(1-\sigma_{2}\right){\bar{C}}_{2}$, $\alpha_{2}=\omega_{22}\left(1-\sigma_{1}\right){\bar{C}}_{1}$, $\alpha_{3}=\omega_{21}\left(1-\sigma_{1}\right){\bar{C}}_{1}$, $\alpha_{4}=\omega_{11}\left(1-\sigma_{2}\right){\bar{C}}_{2}$, $\gamma =\omega_{11}\omega_{22}-\omega_{12}\omega_{21}$, $R_{12}=\left[\omega_{21}\left(1-\sigma_{2}\right)-\omega_{22}\left(1-\sigma_{1}\right)\right]\gamma^{-1}$, $R_{13}=\left[\omega_{12}\left(1-\sigma_{1}\right)-\omega_{11}\left(1-\sigma_{2}\right)\right]\gamma^{-1}$, $R_{21}=\left[\omega_{12}\left(1-\sigma_{2}\right){\bar{C}}_{2}-\omega_{22}\left(1-\sigma_{1}\right){\bar{C}}_{1}\right]\gamma^{-1}$, $R_{22}=\omega_{22}\gamma^{-1}{\bar{C}}_{1}{}^{-1}$, $R_{23}=-\omega_{12}\gamma^{-1}{\bar{C}}_{1}{}^{-1}$, $R_{31}=\left[\omega_{21}\left(1-\sigma_{1}\right){\bar{C}}_{1}-\omega_{11}\left(1-\sigma_{2}\right){\bar{C}}_{2}\right]\gamma^{-1}$, $R_{32}=-\omega_{21}\gamma^{-1}{\bar{C}}_{2}{}^{-1}$, $R_{33}=\omega_{11}\gamma^{-1}{\bar{C}}_{2}{}^{-1}$.

Besides the determinant of matrix [*R*] is given by the relationship:

(8) $$det\;\left[R\right]=\frac{1}{L_{p}{\bar{C}}_{1}{\bar{C}}_{2}\left(\omega_{11}\omega_{22}-\omega_{12}\omega_{21}\right)}\;\;R_{det}\;$$

As in the case of Equation (6), the case of $R_{det}$ = 0 is excluded, because in order for the denominator of Equation (8) to be different from zero, the condition $\omega_{11}\omega_{22}\neq \omega_{12}\omega_{21}$ must be fulfilled. If $\omega_{11}\omega_{22}>\omega_{12}\omega_{21}$ then $R_{det}^{r}$ > 0, and if $\omega_{11}\omega_{22}<\omega_{12}\omega_{21}$ then $R_{det}$ < 0.

The coefficients *R*_11_, *R*_12_, *R*_13_, *R*_21_, *R*_22_, *R*_23_, *R*_31_, *R*_32_ and *R*_33_ occurring in the matrix [*R*] we call Peusner’s coefficients and matrix [*R*]—*R* form of the matrix of Peusner’s coefficients. According to the principles of network thermodynamic [15] in the above equation, symmetry of non-diagonal coefficients (*R_ij_* = *R_ji_ i* ≠ *j*) is not required. In the case considered above for non-diagonal coefficients, we have *R*_12_ = *R*_21_, *R*_13_ = *R*_31_ only when *ω*_12_ = *ω*_21_. Besides, from Equation (7) it results that *R*_23_ = *R*_32_ only when *ω*_12_${\bar{C}}_{1}$ = *ω*_21_ ${\bar{C}}_{2}$.

In order to show the relations between coefficients $R_{ij}^{r}$ and *R_ij_* and between determinants of matrixes [$R^{r}$] and [R] for A and B configurations of the membrane system (*r* = A, B) we calculate using Equations (4)–(7) the expressions:

(9) $$\xi_{ij}=\frac{R_{ij}^{A}\;-\;R_{ij}^{B}}{R_{ij}}$$

(10) $$\xi_{det}=\frac{R_{det}^{A}-R_{det}^{B}}{R_{det}}$$

The values of coefficients *ξ_ij_* and *ξ_det_* show the influence of CP and natural convection (NC) on the membrane transport. These coefficients are a measure of the distance of convective processes from the critical state (non-convection). Assuming that the coefficients $R_{ij}^{A}$, $R_{ij}^{B}$, $R_{ij}$, $R_{det}^{A}$, $R_{det}^{B}$, $\xi_{ij}$ and $\xi_{det}$ have the same sign, on the basis of Equations (9) and (10), we can write the criteria listed in Table 1.

In order to show the relationship between coefficients $R_{ij}$, $R_{ji}$, $R_{ii}$ and $R_{jj}$ and coefficients $R_{ij}^{r}$, $R_{ji}^{r}$, $R_{ii}^{r}$ and $\;R_{jj}^{r}$ for A and B configurations of membrane system we will calculate the Kedem–Caplan–Peusner (KCP) degree of coupling $r_{ij}$ and $r_{ij}^{r}$ in which *i*, *j* ∈ {1, 2, 3}, superscript *r* = A, B, using Equations (5), (7), (11) and (12) [19,20]. The expressions for these coefficients take the following forms:

(11) $$r_{ij}^{r}=-\frac{R_{ij}^{r}}{\sqrt{R_{ii}^{r}R_{jj}^{r}}}$$

(12) $$r_{ij}=-\frac{R_{ij}}{\sqrt{R_{ii}R_{jj}}}$$

The second law of thermodynamics imposes the conditions $R_{ii}^{r}R_{jj}^{r}$ ≥ ${\left(R_{ij}^{r}\right)}^{2}$ and $R_{ii}^{r}R_{jj}^{r}$ ≥ ${\left(R_{ji}^{r}\right)}^{2}$ which means that $r_{ij}^{r}$ and $r_{ji}^{r}$ is limited by the relation −1 ≤ $r_{ij}^{r}$, $r_{ji}^{r}$ ≤ +1. For ternary solutions, taking into consideration Equations (5) and (11) and (7) and (12) we get: $r_{12}^{r}$ ≠ $r_{21}^{r}$, $r_{13}^{r}$ ≠ $r_{31}^{r}$, $r_{23}$ ≠ $r_{32}$ and $r_{23}^{r}$ ≠ $r_{32}^{r}$. This shows that for conditions of CP, Onsager’s reciprocal relations are not satisfied. 

The ${\left(e_{ij}\right)}_{r}$ and ${\left(e_{ij}^{r}\right)}_{r}$ coefficients can be used to evaluate of energy conversion efficiency by means of the Kedem–Caplan–Peusner coefficient, which can be written in the form:

(13) $${\left(e_{ij}^{r}\right)}_{r}=\frac{{\left(r_{ji}^{r}\right)}^{2}}{{\left(1+\sqrt{1-r_{ij}^{r}r_{ji}^{r}}\right)}^{2}}=\frac{{\left(R_{ji}^{r}\right)}^{2}}{R_{ii}^{r}R_{jj}^{r}{\left(1+R_{ii}^{r}R_{jj}^{r}\sqrt{R_{ii}^{r}R_{jj}^{r}-R_{ij}^{r}R_{ji}^{r}}\right)}^{2}}$$

(14) $${\left(e_{ij}\right)}_{r}=\frac{{\left(r_{ji}\right)}^{2}}{{\left(1+\sqrt{1-r_{ij}r_{ji}}\right)}^{2}}=\frac{{\left(R_{ji}\right)}^{2}}{R_{ii}R_{jj}{\left(1+R_{ii}R_{jj}\sqrt{R_{ii}R_{jj}-R_{ij}R_{ji}}\right)}^{2}}$$

Peusner proposed the “super *Q_R_*”—coupling parameter, defined by the following expression [15,21,22]:

(15) $$Q_{R}^{r}=\frac{R_{ij}^{r}R_{ji}^{r}}{2R_{ii}^{r}R_{jj}^{r}-R_{ij}^{r}R_{ji}^{r}}=\frac{r_{ij}^{r}r_{ji}^{r}}{2-r_{ij}^{r}r_{ji}^{r}}$$

(16) $$Q_{R}=\frac{R_{ij}R_{ji}}{2R_{ii}R_{jj}-R_{ij}R_{ji}}=\frac{r_{ij}r_{ji}}{2-r_{ij}r_{ji}}$$

## 3. Results and Discussion

For ternary solutions, the coefficients $R_{ij}^{r}$, *R_ij_*, (*i*, *j* ∈ {1, 2, 3}, *r* = A, B) and determinant of matrix of these coefficients *det* [*R^r^*] were calculated for polymer membrane Nephrophan (VEB Filmfabrik, Wolfen, Germany) and glucose solutions in aqueous solution of ethanol using Equations (2)–(16). Nephrophan is a microporous, highly hydrophilic membrane made of cellulose acetate (cello- triacetate (OCO-CH_3_)_n_). The glucose concentration was marked by Index “1” and the ethanol concentration by Index “2”. The concentration of Substance “1” in Chamber (h) take values from *C*_1*h*_ = 1 mol/m^3^ to *C*_1*h*_ = 101 mol/m^3^. In turn, concentration of a Substance “2” in Chamber (h) was constant and amounted to *C*_2*h*_ = 201 mol/m^3^. The concentrations of both components in the chamber (l) were established and amounted to *C*_1*l*_ = *C*_2*l*_ = 1 mol m^−3^. In expressions under Equation (2) which describe the matrix coefficients $R_{11}^{r}$, $R_{12}^{r}$, $R_{13}^{r}$, $R_{21}^{r}$, $R_{22}^{r}$, $R_{23}^{r}$, $R_{31}^{r}$, $R_{32}^{r}$ and $R_{33}^{r}$ which are the coefficients that describe transport properties of membrane (*L_p_*, *σ*_1_, *σ*_2_, *ω*_11_, *ω*_22_, *ω*_21_ and *ω*_12_), average concentrations of Solutions “1” and “2” in the membrane (${\bar{C}}_{1}$, ${\bar{C}}_{2}$) and CP coefficients ($\zeta_{p}^{r}$, $\zeta_{a1}^{r}$, $\zeta_{a2}^{r}$, $\zeta_{v1}^{r}$, $\zeta_{s11}^{r}$, $\zeta_{s12}^{r}$, $\zeta_{v2}^{r}$, $\zeta_{s22}^{r}$ and $\zeta_{s21}^{r}$). For Nephrophan membrane and aqueous solutions of glucose and ethanol the following conditions are fulfilled: $\zeta_{p}^{r}$ = $\zeta_{a1}^{r}$ = $\zeta_{a2}^{r}$ = 1, $\zeta_{v1}^{r}$ = $\zeta_{s11}^{r}$ = $\zeta_{s12}^{r}$ = $\zeta_{1}^{r}$ and $\zeta_{v2}^{r}$ = $\zeta_{s22}^{r}$ = $\zeta_{s21}^{r}$ = $\zeta_{2}^{r}$ [42]. The coefficients describing transport properties of membrane, e.g., hydraulic permeability (*L_p_*), reflection (*σ*_1_, *σ*_2_) and diffusive permeability (*ω*_11_, *ω*_22_, *ω*_21_, *ω*_12_) were appointed in the conditions of uniform stirring of solutions separated by membrane in series of independent experiments according with the procedure described in the paper [11]. For Nephrophan, membrane values of these coefficients are independent on solution concentration and amount to *L_p_* = 4.9 × 10^−12^ m^3^/Ns, *σ*_1_ = 0.068, *σ*_2_ = 0.025, *ω*_11_ = 0.8 × 10^−9^ mol/Ns, *ω*_12_ = 0.81 × 10^−13^ mol/Ns, *ω*_22_ = 1.43 × 10^−9^ mol/Ns and *ω*_21_ = 1.63 × 10^−12^ mol/Ns [33].

### 3.1. Concentration Dependencies of Coefficients $\zeta_{i}^{r}$ and $\rho^{r}$

**Figure 2:**
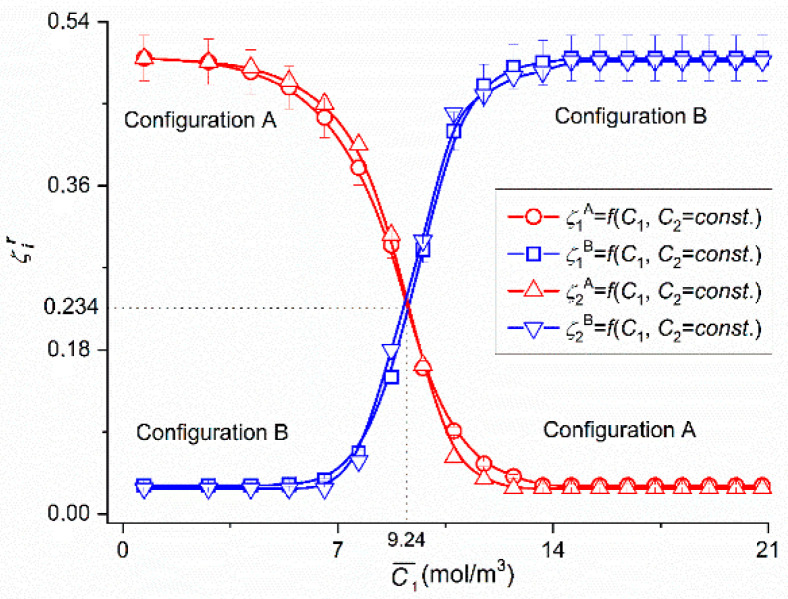
Dependencies of concentration polarization coefficient ($\zeta_{i}^{r}$) on glucose concentration in 201 mol/m^3^ aqueous ethanol solution for Configurations A and B of the single-membrane system.

**Figure 3:**
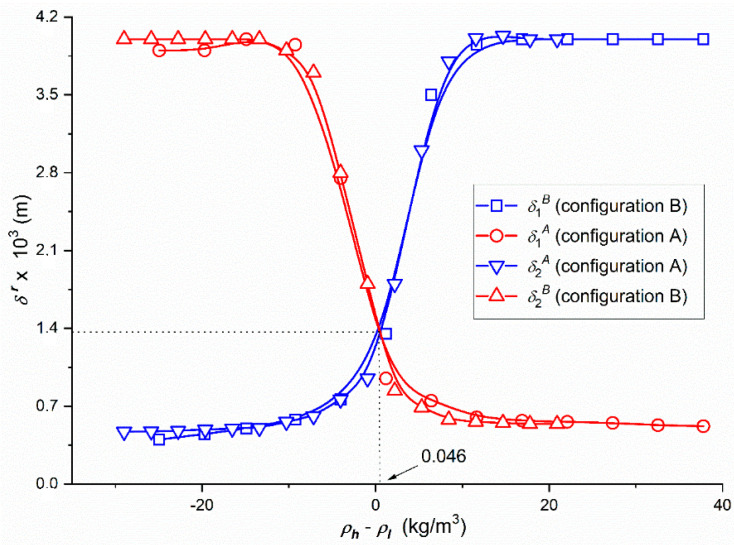
Dependencies of the thickness of concentration boundary layers (*δ^r^*) in Configurations A (*r = A*) and B (*r = B*) of the membrane system on density difference (*ρ_h_*–*ρ_l_*) of glucose concentration in 201 mol/m^3^ aqueous ethanol solutions.

In Figure 2, the experimental dependencies $\zeta_{i}^{r}$ = *f*(${\bar{C}}_{1}$, $\;{\bar{C}}_{2}$ = 37.71 mol/m^3^), (*i* = 1 or 2 and *r* = A or B) were presented for glucose solutions in 201 mol m^−3^ aqueous solution of ethanol taken from our previous paper [42]. The dependences $\rho^{r}$ = *f*(${\bar{C}}_{1}$, $\;{\bar{C}}_{2}\;$ = 37.71 mol/m^3^), (*i* = 1 or 2 and *r* = A or B) presented in Figure 3 were calculated on the basis of Equation (1) and the results shown in Figure 2. The points (○,△) were obtained for Configuration A and points (□,▽) for Configuration B of single-membrane system.

Figure 2 shows that in the case of Configuration A for 0 < ${\bar{C}}_{1}\;$ ≤ 4 mol/m^3^, $\zeta_{1}^{A}$ = $\zeta_{2}^{A}$ = 0.5 = constant and for 4 mol/m^3^ < ${\bar{C}}_{1}\;$ ≤ 12.72 mol/m^3^ the values of coefficients $\zeta_{1}^{A}$ and $\zeta_{2}^{A}$ decrease nonlinearly and for ${\bar{C}}_{1}\;$ > 12.72 mol/m^3^ reach constant value equal respectively to $\zeta_{1}^{A}$ = $\zeta_{2}^{A}$ = 0.03. In the case of Configuration B for 0 < ${\bar{C}}_{1}$ ≤ 5.41 mol/m^3^, $\zeta_{1}^{B}$ = $\zeta_{2}^{B}$ = 0.03 = constant, and for 5.41 mol/m^3^ <${\bar{\;C}}_{1}$ ≤ 12.72 mol/m^3^ the values of coefficients $\zeta_{1}^{A}$ and $\zeta_{2}^{A}$ increase and for ${\bar{C}}_{1}$ > 12.72 mol/m^3^ reach constant value equal respectively to $\zeta_{1}^{A}$ = $\zeta_{2}^{A}$ = 0.5. The results presented in this figure show that 0.5 ≥ $\zeta_{1}^{A}$ ≥ 0.03 and 0.03 ≤ $\zeta_{1}^{B}$ ≤ 0.5. This notation indicates that for the same values ${\bar{C}}_{1}$ and ${\bar{C}}_{2}$ the value of coefficient $\zeta_{1}^{A}$ decreases from 0.5 to 0.03 and coefficient $\zeta_{1}^{B}$ increases from 0.03 to 0.5.

Figure 3 shows that in Configuration A for −30 kg/m^−3^ < $\Delta \rho =\rho_{h}-\rho_{l}\;$ ≤ −8.5 kg/m^−3^, $\delta^{A}$ = 3.95 × 10^−3^ m = constant and for −8.5 kg/m^−3^ < Δρ  ≤ 7.9 kg/m^−3^ the values of coefficients $\delta^{A}$ decrease nonlinearly and for Δρ  > 7.9 kg/m^−3^ reach constant value equal respectively to $\delta^{A}$ = 0.52 × 10^−3^ m = constant. For −30 kg/m^−3^ < Δρ  ≤ −8.5 kg/m^−3^, $\delta^{B}$ = 0.52 × 10^−3^ m = constant and for –8.5 kg/m^–3^ < Δρ  ≤ 7.9 kg/m^−3^ the values of coefficients $\delta^{B}$ decrease nonlinearly and for Δρ  > 7.9 kg/m^−3^ reach constant value equal respectively to $\delta^{B}$ = 3.95 × 10^−3^ m = const. The results presented in Figure 3 show that 3.95 × 10^−3^ m ≥ $\delta^{A}$ ≥ 0.52 × 10^−3^ m and 0.52 × 10^−3^ m ≤ $\delta^{B}$ ≤ 3.95 × 10^−3^ m. This notation indicates that for the same values Δρ the value of coefficient $\delta^{A}$ decreases from 3.95 × 10^−3^ m to 0.52 × 10^−3^ m and coefficient $\delta^{B}$ increases from 0.52 × 10^−3^ m to 3.95 × 10^−3^ m.

In addition, it can be seen from the Figure 2 and Figure 3 that for ${\bar{C}}_{1}\;$ < 9.24 mol/m^3^ and Δρ  ≤ 0.046 kg/m^−3^ in Configuration A, the complex of CBLs is hydrodynamically unstable and in Configuration B—hydrodynamically stable, because the solutions of ethanol prevailing over glucose are under the membrane, and for that case the solution density under the membrane is lower than the solution density over the membrane. In Configuration B, the complex of CBLs is stable because density of the solution under the membrane is greater than the solution above the membrane. In turn for ${\bar{C}}_{1}\;$ > 9.24 mol/m^3^ and Δρ  > 0.046 kg/m^−3^ in Configuration A, the complex of CBLs is hydrodynamically stable, and in Configuration B—hydrodynamically unstable due to the fact that in solutions separated by the membrane, glucose concentration is greater than ethanol and density of solution under the membrane is greater than the solution over the membrane. In Configuration B, the complex of CBLs is unstable because density of the solution under the membrane is smaller than the solution above the membrane. This causes the convection movements vertically downward. For ${\bar{C}}_{1}$ = 9.24 mol/m^3^ and Δρ  = 0.046 kg/m^−3^ the CBL_S_ complex is independent of the membrane system configuration and therefore $\zeta_{1}^{A}$ = $\zeta_{1}^{B}$ = 0.234 and $\delta^{A}$ = $\delta^{B}$ = 1.3 × 10^−3^ m. In Configuration A, a non-convective state occurs, when the density of the solution in the compartment above the membrane is higher than density of the solution in the compartment under the membrane. In Configuration A natural convection occurs when *ρ_l_* > $\rho_{e}^{A}$, $\rho_{i}^{A}$ > *ρ_h_* and $\rho_{e}^{A}$ > $\rho_{i}^{A}\;$ and is directed vertically upwards. On the other hand, in Configuration B, a natural convection occurs when *ρ_l_* < $\rho_{e}^{B}$, $\rho_{i}^{B}$ < *ρ_h_* and $\rho_{e}^{B}\;$ < $\rho_{ei}^{B}$ and is directed vertically downwards [38]. Natural convection allows it to increase the value fluxes of $J_{vk}^{r}$ and $J_{k}^{r}$.

### 3.2. Concentration Dependencies of Coefficients $R_{ij}^{r}$, $R_{ij}$, $R_{det}^{r}$ and $R_{det}$

**Figure 4:**
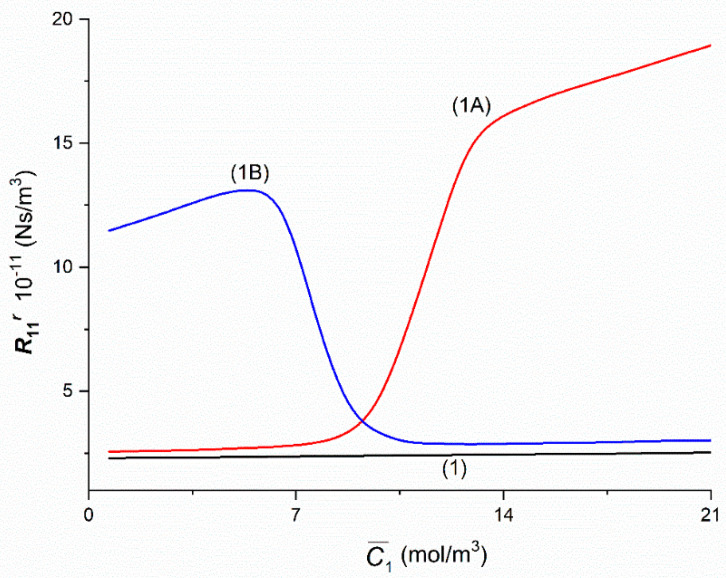
The graphic illustration of the dependences: $R_{ij}^{r}$ = *f*(${\bar{C}}_{1}$, $\;{\bar{C}}_{2}\;$ = 37.71 mol/m^3^), (*i*, *j* ∈ {1, 2, 3} and *r* = A, B) for the glucose in aqueous ethanol solution in conditions of concentration polarization for Configurations A and B of the membrane system: Curve 1A—for $R_{11}^{A}$, Curve 1B—for $R_{11}^{B}$ and Line 1—for *R*_11_.

**Figure 5:**
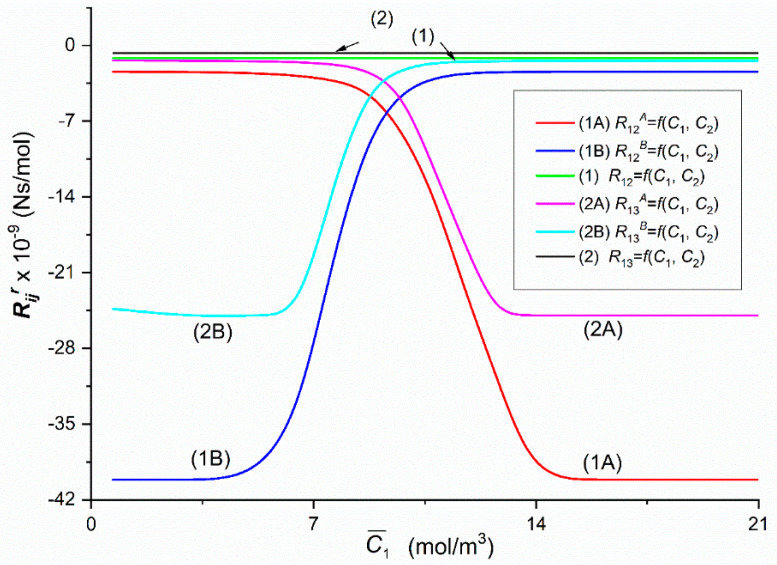
The graphic illustration of the dependences: $R_{ij}^{r}$ = *f*(${\bar{C}}_{1}$, $\;{\bar{C}}_{2}\;$ = 37.71 mol/m^3^), (*i*, *j* ∈ {1, 2, 3} and *r* = A, B) for the glucose in aqueous ethanol solution in conditions of concentration polarization for Configurations A and B of the membrane system: Curve 1A—for $R_{12}^{A}$, Curve 2A—for $R_{13}^{A}$, Curve 1B—for $R_{12}^{B}$, Curve 2B—for $R_{13}^{B}$, Line 1—for *R*_12_ and Line 2—for *R*_13_.

**Figure 6:**
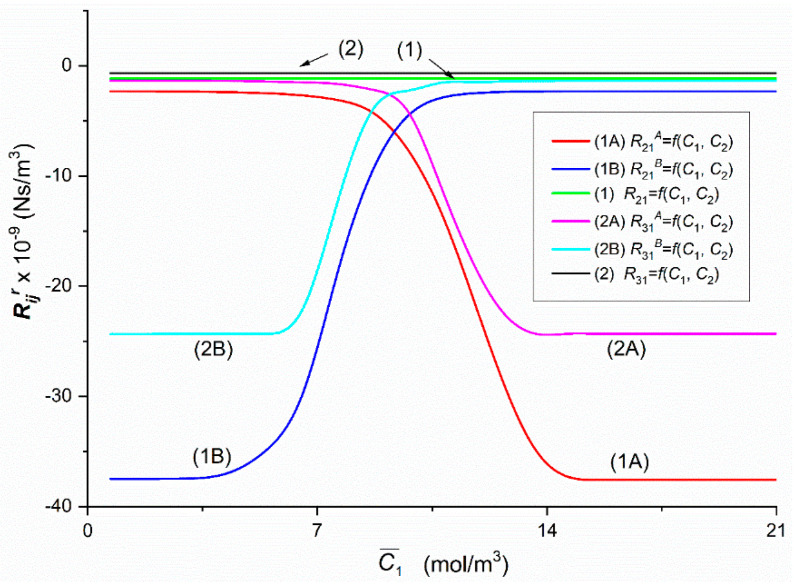
The graphic illustration of the dependences: $R_{ij}^{r}$ = *f*(${\bar{C}}_{1}$, $\;{\bar{C}}_{2}\;$ = 37.71 mol/m^3^), (*i*, *j* ∈ {1, 2, 3} and *r* = A, B) for the glucose in aqueous ethanol solution in conditions of concentration polarization for Configurations A and B of the membrane system: Curve 1A—for $R_{21}^{A}$, Curve 2A—for $R_{31}^{A}$, Curve 1B—for $R_{21}^{B}$, Curve 2B—for $R_{31}^{B}$, Line 1—for *R*_21_ and Line 2—for *R*_31_.

**Figure 7:**
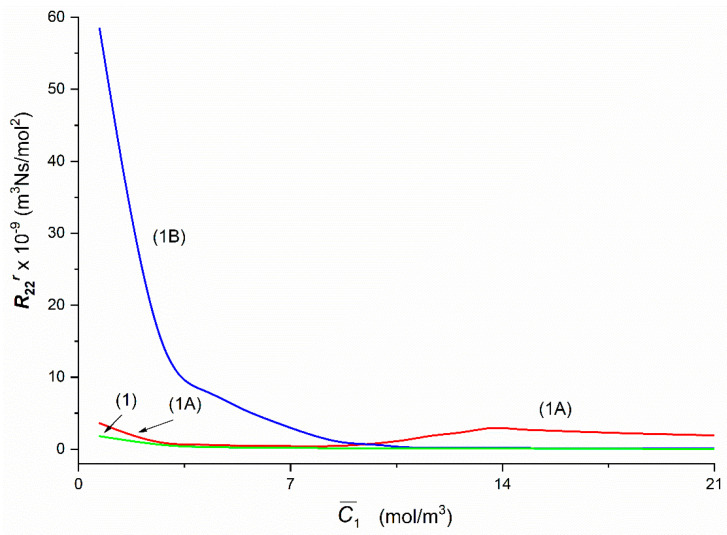
The graphic illustration of the dependences: $R_{22}^{r}$ = *f*(${\bar{C}}_{1}$, $\;{\bar{C}}_{2}\;$ = 37.71 mol/m^3^), (*r* = A, B) for the glucose in aqueous ethanol solution in conditions of concentration polarization for Configurations A and B of the membrane system: Curve 1A—for $R_{22}^{A}$, Curve 1B—for $R_{22}^{B}$ and Line 1—for *R*_22_.

**Figure 8:**
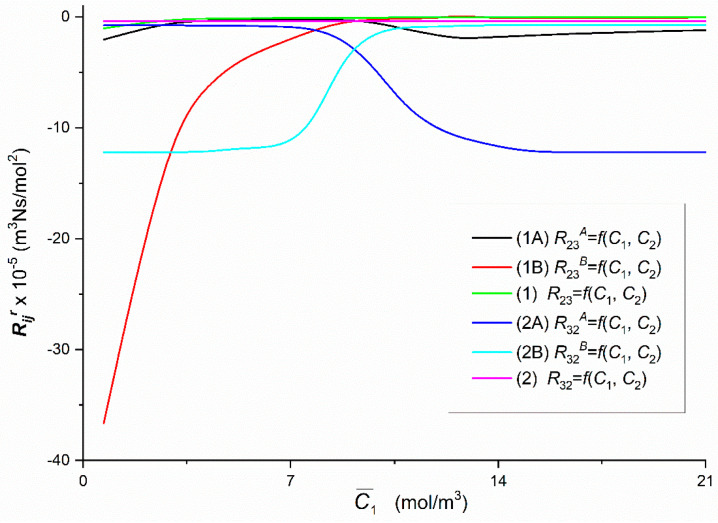
The graphic illustration of the dependences: $R_{ij}^{r}$ = *f*(${\bar{C}}_{1}$, $\;{\bar{C}}_{2}\;$ = 37.71 mol/m^3^), (*i*, *j* ∈ {1, 2, 3} and *r* = A, B) for the glucose in aqueous ethanol solution in conditions of concentration polarization for Configurations A and B of the membrane system: Curve 1A—for $R_{23}^{A}$, Curve 2A—for $R_{32}^{A}$ Curve 1B—for $R_{23}^{B}$ Curve 2B—for $R_{32}^{B}$ Line 1—for *R*_23_ and Line 2—for *R*_32_.

**Figure 9:**
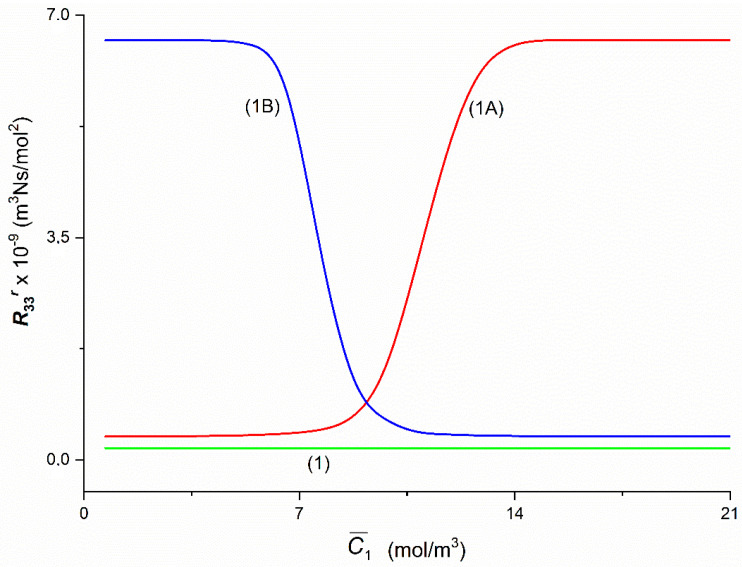
The graphic illustration of the dependence $R_{33}^{r}$ = *f*(${\bar{C}}_{1}$, $\;{\bar{C}}_{2}\;$ = 37.71 mol/m^3^), (*r* = A, B) for the glucose in aqueous ethanol solution in conditions of concentration polarization for Configurations A and B of the membrane system: Curve 1A—for $R_{33}^{A}$, Curve 1B—for $R_{33}^{B}$ and Line 1—for *R*_33_.

**Figure 10:**
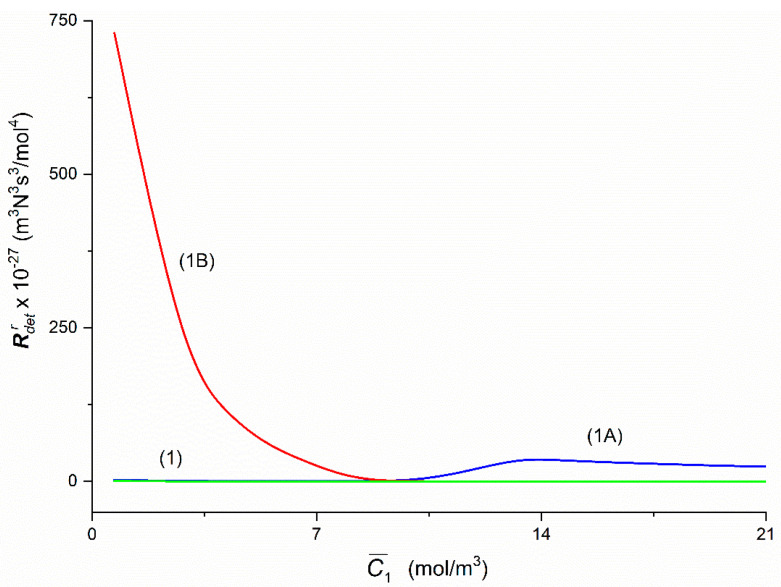
The graphic illustration of the dependence $R_{det}^{r}$ = *f*(${\bar{C}}_{1}$, $\;{\bar{C}}_{2}\;$ = 37.71 mol/m^3^) (*r = A*, *B*) for the glucose in aqueous ethanol solution in conditions of concentration polarization for Configuration A ($R_{det}^{A}$, Curve 1A) and B ($R_{det}^{B}$, Curve 1B) of the membrane system. Line 1 illustrates the dependence $R_{det}$ = *f*(${\bar{C}}_{1}$, $\;{\bar{C}}_{2}\;$ = const.) in conditions of homogeneity of solutions.

To calculate $R_{ij}^{r}$, $R_{ij}$, $R_{det}^{r}$ and $R_{det}$, (*i*, *j* ∈ {1, 2, 3}, *r* = A, B), based on Equations (5)–(8) respectively, the characteristics $\zeta_{1}^{r}=f({\bar{C}}_{1},\;{\bar{C}}_{2}$ = 37.71 mol/m^3^) and $\zeta_{2}^{r}=f({\bar{C}}_{1},\;{\bar{C}}_{2}$ = 37.71 mol/m^3^) presented in Figure 2 and following data: *L_p_* = 4.9 × 10^–12^ m^3^/Ns, *σ*_1_ = 0.068, *σ*_2_ = 0.025, *ω*_11_ = 0.8 × 10^−9^ mol/Ns, *ω*_12_ = 0.81 × 10^−13^ mol/Ns, *ω*_22_ = 1.43 × 10^−9^ mol/Ns, *ω*_21_ = 1.63 × 10^−12^ mol/Ns, ${\bar{C}}_{1}\;$ = 2.79 ÷ 21.67 mol/m^3^ and ${\bar{C}}_{2}\;$ = 37.71 mol/m^3^ were used. The results of calculating these coefficients are presented in Figure 4, Figure 5, Figure 6, Figure 7, Figure 8, Figure 9 and Figure 10.

The Graphs 1A and 1B in Figure 4 illustrating the dependencies $R_{11}^{A}$ = *f*(${\bar{C}}_{1}$, $\;{\bar{C}}_{2}\;$ = 37.71 mol/m^3^) and $R_{11}^{B}$ = *f*(${\bar{C}}_{1}$, $\;{\bar{C}}_{2}\;$ = 37.71 mol/m^3^) were obtained for the Configurations A and B of the membrane system. The value of coefficient $R_{11}^{A}$ increases initially nonlinearly from $R_{11}^{A}$ = 2.57 × 10^11^ Ns/m^3^ (for ${\bar{C}}_{1}\;$ = 1.44 mol/m^3^) to $R_{11}^{A}\;$ = 2.89 × 10^11^ Ns/m^3^ (for ${\bar{C}}_{1}$ = 7.56 mol/m^3^) and next increases nonlinearly to $R_{11}^{A}$ = 16.45 × 10^11^ Ns/m^3^ (for ${\bar{C}}_{1}$ = 14.59 mol/m^3^). For ${\bar{C}}_{1}\;$ > 16.45 mol/m^3^ $R_{11}^{A}$ increases approximately linearly and for ${\bar{C}}_{1}$ = 21.67 mol/m^3^ and ${\bar{C}}_{2}\;$ = 37.71 mol/m^3^ achieves the value $R_{11}^{A}$ = 19.2 × 10^11^ Ns/m^3^. The value of coefficient $R_{11}^{B}$ initially increases linearly from $R_{11}^{B}$ = 11.47 × 10^11^ Ns/m^3^ (for ${\bar{C}}_{1}\;$ = 1.44 mol/m^3^) to $R_{11}^{A}$ = 13.22 × 10^11^ Ns/m^3^ (for ${\bar{C}}_{1}\;$ = 5.41 mol/m^3^) and next decreases almost linearly from $R_{11}^{B}$ = 12.86 × 10^11^ Ns/m^3^ (for ${\bar{C}}_{1}$ = 6.57 mol/m^3^) to $R_{11}^{B}$ = 4.14 × 10^11^ Ns/m^3^ (for ${\bar{C}}_{1}\;\;$ = 8.74 mol/m^3^). Besides *R*_11_*^A^*= $R_{11}^{B}$ = 3.67 × 10^11^ Ns/m^3^ (for ${\bar{C}}_{1}$ = 9.24 mol/m^3^). For ${\bar{C}}_{1}\;$ > 12.72 mol/m^3^ $R_{11}^{A}$ increases approximately linearly and for ${\bar{C}}_{1}$ = 21.67 mol/m^3^ achieves the value $R_{11}^{A}$ = 3.04 × 10^11^ Ns/m^3^. For homogeneous solutions $R_{11}^{A}$ = $R_{11}^{B}$ = *R*_11_ increase linearly from *R*_11_ = 2.3 × 10^11^ Ns/m^3^ (for ${\bar{C}}_{1}\;$ = 1.44 mol/m^3^) to *R*_11_ = 2.53 × 10^11^ Ns/m^3^ (for ${\bar{C}}_{1}$ = 21.67 mol/m^3^). Besides, it follows from this figure that for ${\bar{C}}_{1}$ < 9.24 mol/m^3^ $R_{11}^{A}$ < $R_{11}^{B}$ and for ${\bar{C}}_{1}$ > 9.24 mol/m^3^ $R_{11}^{A}$ > $R_{11}^{B}$.

The Graphs 1A, 1B, 2A and 2B illustrating dependencies $R_{12}^{A}$ = *f*(${\bar{C}}_{1}$, $\;{\bar{C}}_{2}\;$ = 37.71 mol/m^3^), $R_{12}^{B}$ = *f*(${\bar{C}}_{1}$, $\;{\bar{C}}_{2}$ = 37.71 mol/m^3^), $R_{21}^{A}$ = *f*(${\bar{C}}_{1}$, $\;{\bar{C}}_{2}\;$ = 37.71 mol/m^3^) and $R_{21}^{B}$ = *f*(${\bar{C}}_{1}$, $\;{\bar{C}}_{2}\;$ = 37.71 mol/m^3^) presented in Figure 5, were obtained suitably for Configurations A and B of the membrane system, respectively. In the case of Configuration A, the value of coefficients $R_{12}^{A}$ and $R_{13}^{A}$ decreases nonlinearly from $R_{12}^{A}$ = −2.41 × 10^9^ Ns/mol and $R_{13}^{A}$ = −1.41 × 10^9^ Ns/mol (for ${\bar{C}}_{1}$ = 5.41 mol/m^3^) to $R_{12}^{A}$ = −39.95 × 10^−9^ Ns/mol (for ${\bar{C}}_{1}$ = 14.59 mol/m^3^) and to $R_{13}^{A}\;$ = −24.76 × 10^9^ Ns/mol (for ${\bar{C}}_{1}$ = 13.66 mol/m^3^). $R_{12}^{A}$ (for ${\bar{C}}_{1}$ ≥ 15.51 mol/m^3^) and $R_{13}^{A}$ (for ${\bar{C}}_{1}$ ≥ 12.72 mol/m^3^) are constant and amounts to $R_{12}^{A}$ = −40.15 × 10^9^ Ns/mol and $R_{13}^{A}$ = −24.95 × 10^9^ Ns/mol, respectively. The value of coefficients $R_{12}^{B}$ and $R_{13}^{B}$ increases nonlinearly from $R_{12}^{B}$ = −40.15 × 10^9^ Ns/mol and $R_{13}^{B}$ = −24.34 × 10^−9^ Ns/mol (for ${\bar{C}}_{1}$ = 0.69 mol/m^3^) to $R_{12}^{B}\;$ = −2.46 × 10^9^ Ns/mol and $R_{13}^{B}$ = $R_{21}^{B}$ = −1.43 × 10^9^ Ns/mol (for ${\bar{C}}_{1}$ = 12.72 mol/m^3^). For ${\bar{C}}_{1}$ > 13.66 mol/m^3^, $R_{12}^{B}$ and $R_{21}^{B}$ are constant and amounts to $R_{12}^{B}$ = −2.41 × 10^−9^ Ns/mol and $R_{13}^{B}$ = −1.39 × 10^9^ Ns/mol. For ${\bar{C}}_{1}$ = 9.24 mol/m^3^ and ${\bar{C}}_{2}$ = 37.71 mol/m^3^ $R_{12}^{A}$ = $R_{12}^{B}$ = −6.0 × 10^9^ Ns/mol and $R_{13}^{A}$ = $R_{13}^{B}$ = −3.0 × 10^9^ Ns/mol. Besides, for ${\bar{C}}_{1}$ < 9.24 mol/m^3^ $R_{12}^{A}$ > $R_{12}^{B}$ and $R_{13}^{A}$ > $R_{13}^{B}$. For ${\bar{C}}_{1}$ > 9.24 mol/m^3^ $R_{12}^{A}$ < $R_{12}^{B}$ and $R_{13}^{A}$ < $R_{13}^{B}$. For homogeneous solutions $R_{12}^{A}\;$ = $R_{12}^{B}$ = *R*_12_ = −1.16 × 10^9^ Ns/mol < $R_{13}^{A}$ = $R_{13}^{B}$ = *R*_13_ = −0.68 × 10^9^ Ns/mol in whole range of studied ${\bar{C}}_{1}$ (Lines 1 and 2).

The Graphs 1A, 1B, 2A and 2B present dependencies $R_{12}^{A}$ = *f*(${\bar{C}}_{1}$, $\;{\bar{C}}_{2}\;$ = 37.71 mol/m^3^), $R_{12}^{B}$ = *f*(${\bar{C}}_{1}$, $\;{\bar{C}}_{2}$ = 37.71 mol/m^3^), $R_{21}^{A}$ = *f*(${\bar{C}}_{1}$, $\;{\bar{C}}_{2}\;$ = 37.71 mol/m^3^) and $R_{21}^{B}$ = *f*(${\bar{C}}_{1}$, $\;{\bar{C}}_{2}\;$ = 37.71 mol/m^3^) presented in Figure 5, were obtained suitably for Configurations A and B of the membrane system, respectively. In the case of Configuration A, the value of coefficients $R_{12}^{A}$ and $R_{13}^{A}$ decreases nonlinearly from $R_{12}^{A}$ = −2.41 × 10^9^ Ns/mol and $R_{13}^{A}$ = −1.41 × 10^9^ Ns/mol (for ${\bar{C}}_{1}$ = 5.41 mol/m^3^) to $R_{12}^{A}$ = −39.95 × 10^−9^ Ns/mol (for ${\bar{C}}_{1}$ = 14.59 mol/m^3^) and to $R_{13}^{A}\;$ = −24.76 × 10^9^ Ns/mol (for ${\bar{C}}_{1}$ = 13.66 mol/m^3^). $R_{12}^{A}$ (for ${\bar{C}}_{1}$ ≥ 15.51 mol/m^3^) and $R_{13}^{A}$ (for ${\bar{C}}_{1}$ ≥ 12.72 mol/m^3^) are constant and amounts to $R_{12}^{A}$ = −40.15 × 10^9^ Ns/mol and $R_{13}^{A}$ = −24.95 × 10^9^ Ns/mol, respectively. The value of coefficients $R_{12}^{B}$ and $R_{13}^{B}$ increases nonlinearly from $R_{12}^{B}$ = −40.15 × 10^9^ Ns/mol and $R_{13}^{B}$ = −24.34 × 10^−9^ Ns/mol (for ${\bar{C}}_{1}$ = 0.69 mol/m^3^) to $R_{12}^{B}\;$ = −2.46 × 10^9^ Ns/mol and $R_{13}^{B}$ = $R_{21}^{B}$ = −1.43 × 10^9^ Ns/mol (for ${\bar{C}}_{1}$ = 12.72 mol/m^3^). For ${\bar{C}}_{1}$ > 13.66 mol/m^3^, $R_{12}^{B}$ and $R_{21}^{B}$ are constant and amounts to $R_{12}^{B}$ = −2.41 × 10^−9^ Ns/mol and $R_{13}^{B}$ = −1.39 × 10^9^ Ns/mol. For ${\bar{C}}_{1}$ = 9.24 mol/m^3^ and ${\bar{C}}_{2}$ = 37.71 mol/m^3^ $R_{12}^{A}$ = $R_{12}^{B}$ = −6.0 × 10^9^ Ns/mol and $R_{13}^{A}$ = $R_{13}^{B}$ = −3.0 × 10^9^ Ns/mol. Besides, for ${\bar{C}}_{1}$ < 9.24 mol/m^3^ $R_{12}^{A}$ > $R_{12}^{B}$ and $R_{13}^{A}$ > $R_{13}^{B}$. For ${\bar{C}}_{1}$ > 9.24 mol/m^3^ $R_{12}^{A}$ < $R_{12}^{B}$ and $R_{13}^{A}$ < $R_{13}^{B}$. For homogeneous solutions $R_{12}^{A}\;$ = $R_{12}^{B}$ = *R*_12_ = −1.16 × 10^9^ Ns/mol < $R_{13}^{A}$ = $R_{13}^{B}$ = *R*_13_ = −0.68 × 10^9^ Ns/mol in whole range of studied ${\bar{C}}_{1}$ (Lines 1 and 2).

Graphs 1A, 1B, 2A and 2B illustrating dependencies $R_{21}^{A}$ = *f*(${\bar{C}}_{1}$, ${\bar{C}}_{2}$ = 37.71 mol/m^3^), $R_{21}^{B}$ = *f*(${\bar{C}}_{1}$, $\;{\bar{C}}_{2}$ = 37.71 mol/m^3^), $R_{31}^{A}$ = *f*(${\bar{C}}_{1}$, $\;{\bar{C}}_{2}$ = 37.71 mol/m^3^) and $R_{31}^{B}$ = *f*(${\bar{C}}_{1}$, $\;{\bar{C}}_{2}$ = 37.71 mol/m^3^), presented in Figure 6, were obtained suitably for Configurations A and B of the membrane system, respectively. The dependencies shown in this figure are similar to the dependencies shown in Figure 5.

The Graphs 1A and 1B, illustrating the dependencies $R_{22}^{A}$ = *f*(${\bar{C}}_{1}$, $\;{\bar{C}}_{2}$ = 37.71 mol/m^3^) and $R_{22}^{B}$ = *f*(${\bar{C}}_{1}$, $\;{\bar{C}}_{2}$ = 37.71 mol/m^3^), presented in Figure 7, were obtained for the Configurations A and B of the membrane system. Curves 1 and 1B illustrate the dependencies *R*_22_ = *f*(${\bar{C}}_{1}$, $\;{\bar{C}}_{2}$ = 37.71 mol/m^3^) and $R_{22}^{B}$ = *f*(${\bar{C}}_{1}$, $\;{\bar{C}}_{2}$ = 37.71 mol/m^3^) are hyperbolas. In turn, Curve 1A, illustrating the dependence $R_{22}^{A}$ = *f*(${\bar{C}}_{1}$, $\;{\bar{C}}_{2}$), is an irregular curve: initially it decreases nonlinearly from $R_{22}^{A}$ = 3.62 × 10^9^ m^3^Ns/mol^2^ (for ${\bar{C}}_{1}$ = 1.44 mol/m^3^) to $R_{22}^{A}$ = 0.43 × 10^9^ m^3^Ns/mol^2^ (for ${\bar{C}}_{1}$ = 7.68 mol/m^3^) and then grows nonlinearly to $R_{22}^{A}$ = 2.95 × 10^9^ m^3^Ns/mol^2^ (for ${\bar{C}}_{1}$ = 13.66 mol/m^3^). For ${\bar{C}}_{1}$ > 13.66 mol/m^3^ $R_{22}^{A}$ decreases linearly to $R_{22}^{A}$ = 1.86 × 10^9^ m^3^Ns/mol^2^ (for ${\bar{C}}_{1}$ = 21.67 mol/m^3^). In turn for ${\bar{C}}_{1}$ = 9.24 mol/m^3^ $R_{22}^{A}$ = $R_{22}^{B}$ = 0.64 × 10^9^ m^3^Ns/mol^2^, while for ${\bar{C}}_{1}$ < 9.24 mol/m^3^ $R_{22}^{A}$ < $R_{22}^{B}$ and for ${\bar{C}}_{1}$ > 9.24 mol/m^3^ $R_{22}^{A}$ > $R_{22}^{B}$.

Graphs 1A, 1B, 2A and 2B illustrate dependencies $R_{23}^{A}$ = *f*(${\bar{C}}_{1}$, $\;{\bar{C}}_{2}\;$ = 37.71 mol/m^3^ = const.), $R_{23}^{B}$ = *f*(${\bar{C}}_{1}$, $\;{\bar{C}}_{2}\;$ = 37.71 mol/m^3^ = const.), $R_{32}^{A}$ = *f*(${\bar{C}}_{1}$, $\;{\bar{C}}_{2}\;$ = 37.71 mol/m^3^ = const.) and $R_{32}^{B}$ = *f*(${\bar{C}}_{1}$, $\;{\bar{C}}_{2}$ = 7.71 mol/m^3^ = const.) as presented in Figure 8, were obtained suitably for Configurations A and B of the membrane system, respectively. Curves 1, 2 and 1B illustrate dependencies *R*_23_ = *f*(${\bar{C}}_{1}$, $\;{\bar{C}}_{2}$ = 37.71 mol/m^3^ = const.), *R*_32_ = *f*(${\bar{C}}_{1}$, $\;{\bar{C}}_{2}$ = 37.71 mol/m^3^ = const.) and $R_{23}^{B}$ = *f*(${\bar{C}}_{1}$, $\;{\bar{C}}_{2}$ = 37.71 mol/m^3^ = const.) are hyperbolas. In turn, Curve 1A illustrating the dependence $R_{23}^{A}$ = *f*(${\bar{C}}_{1}$, $\;{\bar{C}}_{2}$ = 37.71 mol/m^3^ = const.) is an irregular curve: initially it grows nonlinearly from $R_{23}^{A}$ = −2.05 × 10^5^ m^3^Ns/mol^2^ (for ${\bar{C}}_{1}$ = 1.44 mol/m^3^) to $R_{23}^{A}$ = −0.23 × 10^5^ m^3^Ns/mol^2^ (for ${\bar{C}}_{1}$ = 7.68 mol/m^3^) and then decreases nonlinearly to $R_{23}^{A}$ = −1.99 × 10^5^ m^3^Ns/mol^2^ (for ${\bar{C}}_{1}$ = 13.66 mol/m^3^). For ${\bar{C}}_{1}$ > 12.71 mol/m^3^ $R_{23}^{A}$ increases linearly to $R_{23}^{A}$ = −1.17 × 10^5^ m^3^Ns/mol^2^ (for ${\bar{C}}_{1}$ = 21.67 mol/m^3^). For ${\bar{C}}_{1}$ = 9.24 mol/m^3^, $R_{23}^{A}$ = $R_{23}^{B}$ = −0.38 × 10^5^ m^3^Ns/mol^2^, while for ${\bar{C}}_{1}$ < 9.24 mol/m^3^ $R_{23}^{A}$ > $R_{23}^{B}$ and for ${\bar{C}}_{1}$ > 9.24 mol/m^3^ $R_{23}^{A}$ < $R_{23}^{B}.$ Curves 2A and 2B illustrating respectively the dependence $R_{32}^{A}$ = *f*(${\bar{C}}_{1}$, $\;{\bar{C}}_{2}$) and *R*_32_*^B^*= *f*(${\bar{C}}_{1}$, $\;{\bar{C}}_{2}$) intersect at the coordinates ${\bar{C}}_{1}$ = 9.24 mol/m^3^ and $R_{32}^{A}$ = $R_{32}^{B}$ = −2.66 × 10^5^ m^3^Ns/mol^2^. For ${\bar{C}}_{1}$ < 9.24 mol/m^3^ $R_{32}^{A}$ > $R_{32}^{B}$ and for ${\bar{C}}_{1}$ > 9.24 mol/m^3^ $R_{32}^{A}$ < $R_{32}^{B}$.

Presented in Figure 9, Graphs 1A and 1B illustrating the dependencies $R_{33}^{A}$ = *f*(${\bar{C}}_{1}$, $\;{\bar{C}}_{2}$ = 37.71 mol/m^3^) and $R_{33}^{B}$ = *f*(${\bar{C}}_{1}$, $\;{\bar{C}}_{2}$ = 37.71 mol/m^3^) were obtained for Configurations A and B of the membrane system. The value of coefficient $R_{33}^{A}$ increases nonlinearly from $R_{33}^{A}$ = 0.37 × 10^9^ m^3^Ns/mol^2^ (for ${\bar{C}}_{1}$ = 1.44 mol/m^3^) to $R_{33}^{A}$ = 6.51 × 10^9^ m^3^Ns/mol^2^ (for ${\bar{C}}_{1}$ = 13.66 mol/m^3^). For ${\bar{C}}_{1}$ > 13.66 mol/m^3^ $R_{33}^{A}$ = 6.61 × 10^9^ m^3^Ns/mol^2^ and is constant. The value of coefficient $R_{33}^{B}$ in Configuration B of the membrane system initially is constant and for ${\bar{C}}_{1}$ > 1.44 mol/m^3^ increases nonlinearly from $R_{33}^{B}$ = 6.61 × 10^9^ m^3^Ns/mol^2^ (for ${\bar{C}}_{1}$ = 0.69 mol/m^3^) to $R_{33}^{B}$ = 0.38 × 10^9^ m^3^Ns/mol^2^ (for ${\bar{C}}_{1}$ = 13.66 mol/m^3^) and next achieves constant value $R_{33}^{B}$ = 0.37 × 10^9^ m^3^Ns/mol^2^ (for ${\bar{C}}_{1}$ > 13.66 mol/m^3^). Besides $R_{33}^{A}$ = $R_{33}^{B}$ = 0.82 × 10^9^ m^3^Ns/mol^2^ for ${\bar{C}}_{1}$ = 9.24 mol/m^3^ and ${\bar{C}}_{2}$ = 37.71 mol/m^3^. For homogeneous solutions $R_{33}^{A}$ = $R_{33}^{B}$ = *R*_33_, *R*_33_ = 0.18 × 10^9^ m^3^Ns/mol^2^ (for ${\bar{C}}_{1}$ = 0.69 mol/m^3^). Besides, it follows from this figure that for ${\bar{C}}_{1}$ < 9.24 mol/m^3^ $R_{33}^{A}$ < $R_{33}^{B}$ and for ${\bar{C}}_{1}$ > 9.24 mol/m^3^ $R_{33}^{A}$ > $R_{33}^{B}$.

The curves presented in Figure 2, Figure 3, Figure 4, Figure 5, Figure 6, Figure 7, Figure 8 and Figure 9, marked with a number and letters A or B, show that there are transition points from a linear wave to a non-linear wave or vice versa. It is related to the change of the nature of membrane transport from osmotic-diffusion to osmotic-diffusion-convective, or—inversely. The mechanism of this process is as follows. As the concentration of glucose increases at a given concentration of ethanol, the density of the solution, filling the compartment under the membrane in Configuration B, increases. If the density of this solution is lower than the density of the solution filling the compartment above the membrane, natural convection occurs in Configuration B, which causes destruction of CBLs, increasing driving forces and increasing the value of the coefficient. The addition of glucose stabilizes the layers and finally eliminates natural convection and changes the nature of transport from osmotic-diffusion-convective to osmotic-diffusion. In Configuration A, the process of creating gravitational convection is in the reverse order. This means that in Configuration A we have a transition from non-convective to convective, and in Configuration B—from convective to non-convective states. These transitions have a pseudo-phase transition character.

To calculate coefficients $R_{det}^{r}$ and $R_{det}$ the Equations (6) and (8) were used, respectively. The Graphs 1A and 1B presented in Figure 10 and illustrating the dependencies $R_{det}^{A}$ = *f*(${\bar{C}}_{1}$, $\;{\bar{C}}_{2}$ = 37.71 mol/m^3^ = const.) and $R_{det}^{B}$ = *f*(${\bar{C}}_{1}$, $\;{\bar{C}}_{2}$ = 37.71 mol/m^3^ = const.) were obtained for the Configurations A and B of the membrane system. Curve 1B is hyperbolic. In turn, Curve 1A is an irregular curve: initially it decreases nonlinearly from $R_{det}^{A}$ = 2.53 × 10^27^ m^3^N^3^s^3^/mol^4^ (for ${\bar{C}}_{1}$ = 1.44 mol/m^3^) to $R_{det}^{A}$ = 0.7 × 10^27^ m^3^N^3^s^3^/mol^4^ (for ${\bar{C}}_{1}$ = 7.68 mol/m^3^) and then grows nonlinearly to $R_{det}^{A}$ = 36.86 × 10^27^ m^3^N^3^s^3^/mol^4^ (for ${\bar{C}}_{1}$ = 13.66 mol m^−3^, ${\bar{C}}_{2}$ = 37.71 mol/m^3^). For ${\bar{C}}_{1}$ > 13.66 mol/m^3^ $R_{det}^{A}$ decreases linearly to the value of $R_{det}^{A}$ = 23.24 × 10^27^ m^3^N^3^s^3^/mol^4^ (for ${\bar{C}}_{1}$ = 21.67 mol/m^3^). In turn for ${\bar{C}}_{1}$ = 9.24 mol/m^3^ $R_{det}^{A}$ = $R_{det}^{B}$ = 11.20 × 10^27^ m^3^N^3^s^3^/mol^4^, whereas for ${\bar{C}}_{1}$ < 9.24 mol/m^3^ $R_{det}^{A}$ < $R_{det}^{B}$ and for ${\bar{C}}_{1}$ > 9.24 mol/m^3^ $R_{det}^{A}$ > $R_{det}^{B}$. For homogeneous solutions, $R_{det}^{A}$ = $R_{det}^{B}$ = $R_{det}$ increase linearly from $R_{det}$ = 0.63 × 10^27^ m^3^N^3^s^3^/mol^4^ (for ${\bar{C}}_{1}$ = 1.44 mol/m^3^) to $R_{det}$ = 0.02 × 10^27^ m^3^N^3^s^3^/mol^4^ (for ${\bar{C}}_{1}$ = 21.67 mol/m^3^).

### 3.3. Concentration Dependencies of $\xi_{ij}$ and $\xi_{det}$

**Figure 11:**
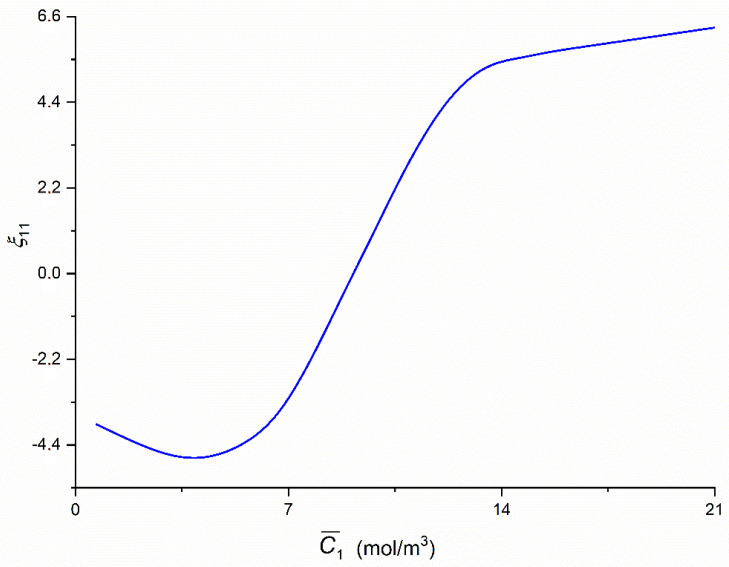
The graphic illustration of the dependence *ξ*_11_ = *f*(${\bar{C}}_{1}$, $\;{\bar{C}}_{2}\;$ = 37.71 mol/m^3^) for the glucose in aqueous ethanol solution.

To calculate coefficients $\xi_{ij}$ = ($R_{ij}^{A}$ − $R_{ij}^{B}$)/$R_{ij}$ and $\xi_{det}$ = (*det* [$R^{A}$] – *det* [$R^{B}$])/*det* [*R*] the Equations (9) and (10) were used, respectively. The graph presented in Figure 11 illustrating the dependencies $\xi_{11}\;$ = *f*(${\bar{C}}_{1}$, $\;{\bar{C}}_{2}$ = 37.71 mol/m^3^) was calculated on the basis of Equation (9). In that case the value of coefficient $\xi_{11}$ initially decreases to $\xi_{11}$ = −4.8 (for ${\bar{C}}_{1}$ = 1.44 mol/m^3^) and next increases nonlinearly to $\xi_{11}$ = 5.41 (for ${\bar{C}}_{1}$ = 13.66 mol/m^3^) and then increases linearly to $\xi_{11}$ = 6.39 (for ${\bar{C}}_{1}$ = 21.67 mol/m^3^). Besides, it follows from this figure that for ${\bar{C}}_{1}$ = 9.24 mol/m^3^ $\xi_{11}$ = 0 and that ${\bar{C}}_{1}$ < 9.24 mol/m^3^ $\xi_{11}$ < 0 and for ${\bar{C}}_{1}$ > 9.24 mol/m^3^ $\xi_{11}$ < 0.

**Figure 12:**
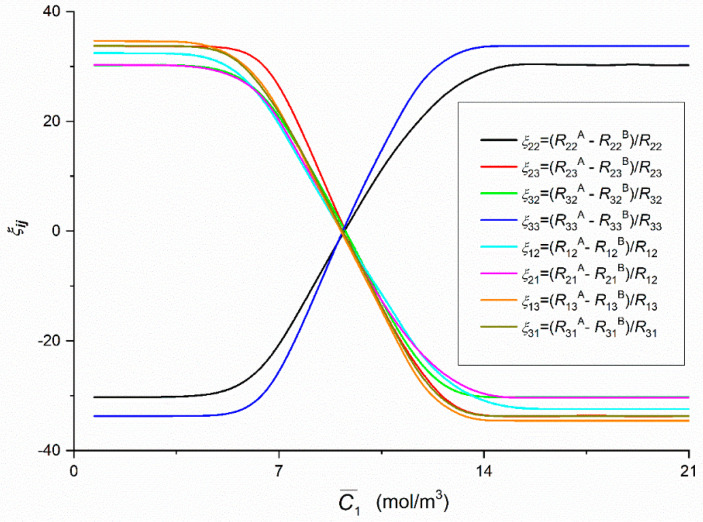
The graphic illustration of the dependence $\xi_{ij}$ = *f*(${\bar{C}}_{1}$, $\;{\bar{C}}_{2}\;$ = 37.71 mol/m^3^) (*i*, *j* ∈ {1, 2, 3} and *r* = A, B).

The graphs presented in Figure 12 which illustrate the dependencies *ξ*_12_ = *f*(${\bar{C}}_{1}$, $\;{\bar{C}}_{2}\;$ = 37.71 mol/m^3^), *ξ*_13_ = *f*(${\bar{C}}_{1}$, $\;{\bar{C}}_{2}\;$ = 37.71 mol/m^3^), *ξ*_21_ = *f*(${\bar{C}}_{1}$, $\;{\bar{C}}_{2}\;$ = 37.71 mol/m^3^), *ξ*_31_ = *f*(${\bar{C}}_{1}$, $\;{\bar{C}}_{2}\;$ = 37.71 mol/m^3^), *ξ*_22_ = *f*(${\bar{C}}_{1}$, $\;{\bar{C}}_{2}\;$ = 37.71 mol/m^3^), *ξ*_32_ = *f*(${\bar{C}}_{1}$, $\;{\bar{C}}_{2}\;$ = 37.71 mol/m^3^), *ξ*_23_ = *f*(${\bar{C}}_{1}$, $\;{\bar{C}}_{2}\;$ = 37.71 mol/m^3^) and *ξ*_33_ = *f*(${\bar{C}}_{1}$, $\;{\bar{C}}_{2}\;$ = 37.71 mol/m^3^) were calculated on the basis of Equation (9). For these graphs, the value of coefficients *ξ*_12_, *ξ*_13_, *ξ*_21_, *ξ*_31_, *ξ*_23_ and *ξ*_32_ decreases nonlinearly (initially slowly and then faster) from *ξ*_12_ > 0 = constant, *ξ*_13_ > 0 = constant, *ξ*_21_ > 0 = constant, *ξ*_31_ > 0 = constant, *ξ*_23_ > 0 = constant and *ξ*_32_ > 0 = constant (*ξ*_21_ < *ξ*_12_ < *ξ*_31_ < *ξ*_13_ < *ξ*_32_ < *ξ*_23_), next *ξ*_12_, *ξ*_13_, *ξ*_21_, *ξ*_31_, *ξ*_23_ and *ξ*_32_ decreases linearly to *ξ*_12_ < 0 = const., *ξ*_13_ < 0 = const, *ξ*_21_ < 0 = const., *ξ*_31_ < 0 = const., *ξ*_23_ < 0 = const. and *ξ*_32_ < 0 = constant (*ξ*_21_ > *ξ*_32_ > *ξ*_12_ > *ξ*_23_ > *ξ*_31_ > *ξ*_13_). It results from this figure that *ξ*_12_ = *ξ*_13_ = *ξ*_21_ = *ξ*_31_ = *ξ*_23_ = *ξ*_32_ = 0 for ${\bar{C}}_{1}$ = 9.24 mol/m^3^. For these graphs the value of coefficients *ξ*_22_ and *ξ*_33_ increases nonlinearly (initially slowly and then faster) from *ξ*_22_ < 0 = constant and *ξ*_33_ < 0 = constant (*ξ*_22_ > *ξ*_33_), next *ξ*_22_ and *ξ*_33_ increases linearly to *ξ*_22_ > 0 = const. and *ξ*_33_ > 0 = constant. Besides, it follows from this figure that for ${\bar{C}}_{1}$ = 9.24 mol m^−3^, *ξ*_22_ = *ξ*_33_ = 0.

**Figure 13:**
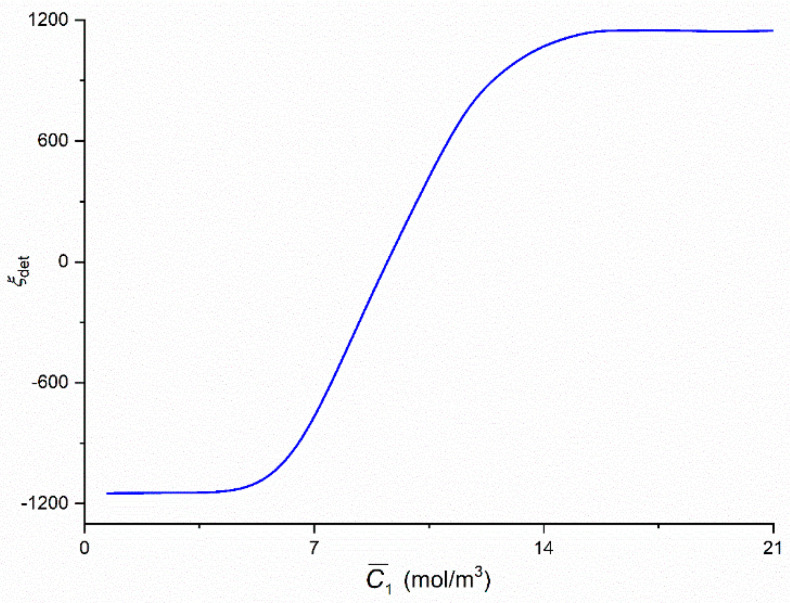
The graphic illustration of the dependence $\xi_{det}$ = *f*(${\bar{C}}_{1}$, $\;{\bar{C}}_{2}\;$ = 37.71 mol/m^3^).

The graph presented in Figure 13 illustrating the dependencies *ξ_det_* = *f*(${\bar{C}}_{1}$, $\;{\bar{C}}_{2}\;$ = 37.71 mol/m^3^ = const.) was calculated on the basis of Equation (10). In the case of this curve the value of coefficient *ξ_det_* initially is constant and amounts *ξ_det_* = −0.034 and next increases nonlinearly to *ξ_det_* = −1148.94 (for ${\bar{C}}_{1}$ = 1.44 mol/m^3^), then increases linearly to *ξ_det_* = 866.38 (for ${\bar{C}}_{1}$ = 12.73 mol/m^3^) and next, nonlinearly to *ξ_det_* = 1148.38 (for ${\bar{C}}_{1}$ ≥ 21.67 mol/m^3^). Besides, it follows from this figure that for ${\bar{C}}_{1}$ = 9.24 mol/m^3^, *ξ_det_* = 0.

In all cases of the dependencies, $R_{ij}^{r}$ = *f*(${\bar{C}}_{1}$, $\;{\bar{C}}_{2}$ = 37.71 mol/m^3^) (*i*, *j* ∈ {1, 2, 3}, *r* = A or B) and $R_{det}^{r}$ = *f*(${\bar{C}}_{1}$, $\;{\bar{C}}_{2}$ = 37.71 mol/m^3^), (*r* = A or B) shown in Figure 5, Figure 6, Figure 7, Figure 8, Figure 9 and Figure 10 show clearly that their values are determined by the hydrodynamic conditions in solutions near membrane which separates ternary non-electrolytes with different concentrations. It means that values of these coefficients in concentration polarization conditions are strongly connected with concentrations ${\bar{C}}_{1}$ and ${\bar{C}}_{2}$ and configuration of the membrane system. In turn, in the case of mechanical stirring of solutions, the values of these coefficients depend only on concentrations ${\bar{C}}_{1}$ and ${\bar{C}}_{2}$. Therefore, for interpretation of calculation results, the combinations of coefficients $R_{ij}^{A}$, $R_{ij}^{B}$ and *R_ij_* (*i*, *j* ∈ {1, 2, 3) of the same indicators and $R_{det}^{A}$, $R_{det}^{B}$ and $R_{det}$ were used. These combinations are presented by Equations (5)–(10). Concentration dependencies of new coefficients facilitate the location of areas differentiated by hydrodynamic conditions in adjacent membrane areas such as diffusion, natural convection-diffusion and natural convection.

Comparison of the results of the tests presented in Figure 4, Figure 5, Figure 6, Figure 7, Figure 8, Figure 9 and Figure 10 and the results presented in Figure 11, Figure 12 and Figure 13 results in the signs of the factors $\xi_{ij}$ and $\xi_{det}$. The results of this comparison are summarized in Table 2.

From the results presented in Figure 4, Figure 5, Figure 6, Figure 7, Figure 8, Figure 9 and Figure 10, it also appears that the $R_{ij}^{r}$ and $R_{det}^{r}$ (*i*, *j* ∈ {1, 2, 3}, *r* = A, B), have different physical significance. The unit of coefficients $R_{11}^{r}\;$, $R_{21}^{r}\;$ and $\;R_{31}^{r}$ is Ns/m^3^. Therefore, they have the character of flow resistance coefficients (hydraulic resistance). In turn, the unit of coefficients $R_{12}^{r}$ i $R_{13}^{r}$ is Ns/mol, what makes them coefficients of flow resistance of dissolved substances (diffusion resistance). The unit of coefficients $R_{22}^{r}$, $R_{23}^{r}$, $R_{32}^{r}$ and $R_{33}^{r}\;$ is m^3^Ns/mol^2^. This unit is a measure of the ratio of diffusion resistance to concentration. The unit of the coefficient $R_{det}^{r}$ is m^3^N^3^s^3^/mol^4^. It corresponds to the ratio of diffusion resistance raised to the power of third and concentration.

### 3.4. Concentration Dependencies of $r_{ij}^{r}$, $r_{ij}$, $e_{ij}^{r}$, $e_{ij}$, $Q_{R}^{r}$ and $Q_{R}$

**Figure 14:**
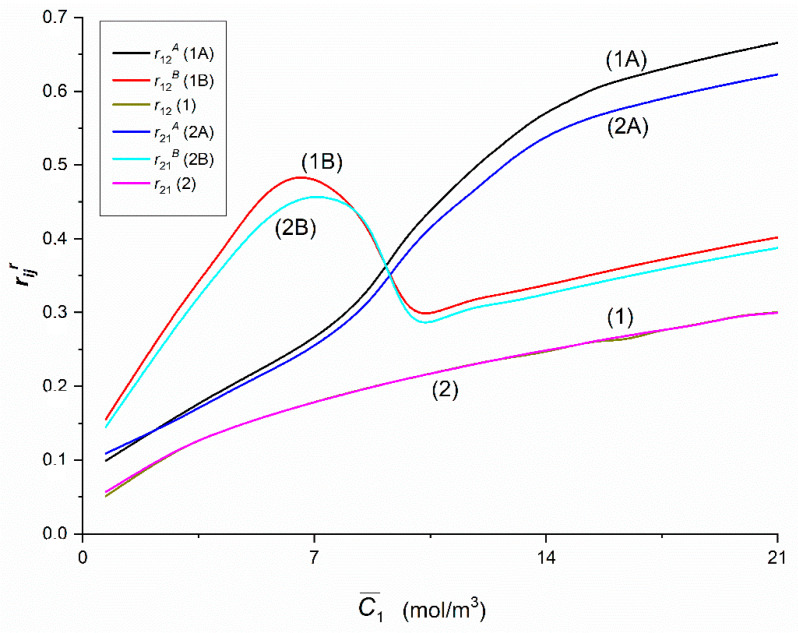
The $r_{ij}^{r}$ and $r_{ij}$ (*i*, *j* ∈ {1, 2}, *r* = A, B) coefficients as functions of glucose concentration.

**Figure 15:**
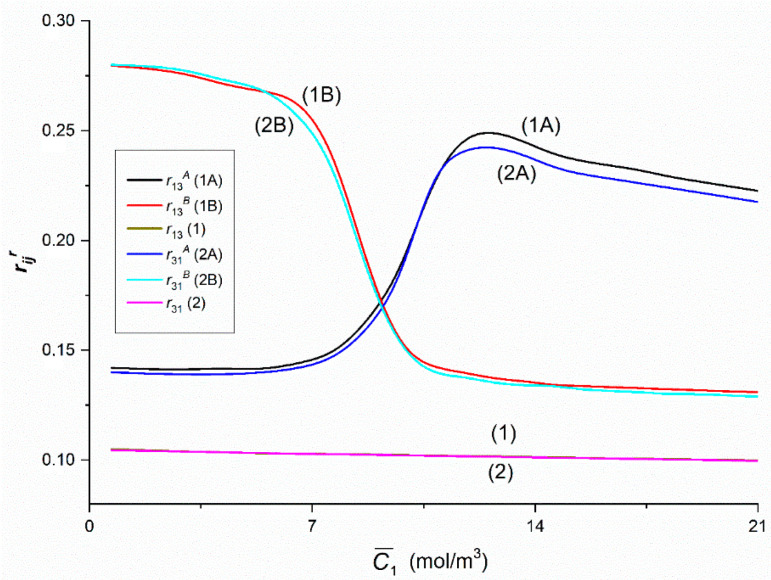
The $r_{ij}^{r}$ and $r_{ij}$ (*i*, *j* ∈ {1, 3}, *r* = A, B) coefficients as functions of glucose concentration.

**Figure 16:**
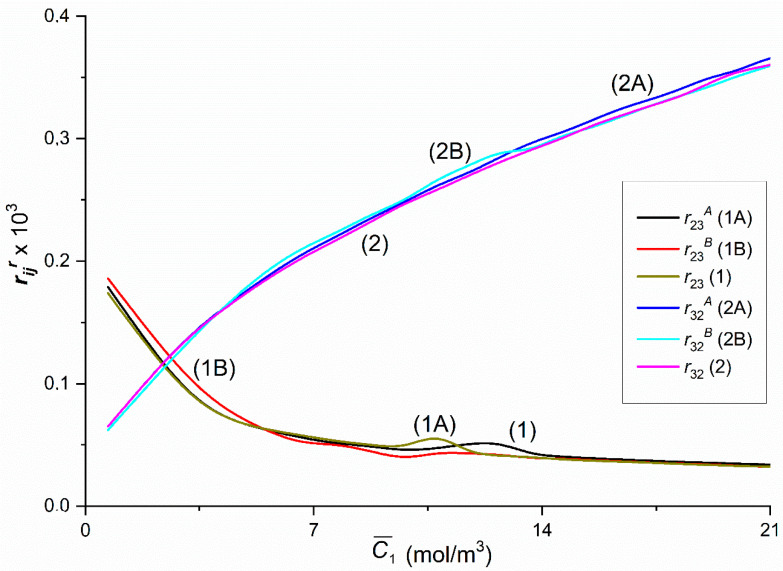
The $r_{ij}^{r}$ and $r_{ij}$ (*i*, *j* ∈ {2, 3}, *r* = A, B) coefficients as functions of glucose concentration.

Figure 14, Figure 15 and Figure 16 show the dependences $r_{ij}^{r}$ = *f*(${\bar{C}}_{1}$, $\;{\bar{C}}_{2}$ = 37.71 mol/m^3^) and $r_{ij}$ = *f*(${\bar{C}}_{1}$, $\;{\bar{C}}_{2}$ = 37.71 mol/m^3^), (*i*, *j* ∈ {1, 2, 3} and *r* = A, B) calculated on the basis of Equations (11) and (12) and data presented in Figure 4, Figure 5, Figure 6, Figure 7, Figure 8 and Figure 9. Figure 14 shows that Curves 1A and 1B intersect at a point with coordinates: $r_{12}^{A}$ = $r_{12}^{B}$ = 0.36 and ${\bar{C}}_{1}\;$ = 9.15 mol/m^3^, and the Curves 2A and 2B—at a point with coordinates: $r_{21}^{A}$ = $r_{21}^{B}$ = 0.35 and ${\bar{C}}_{1}$ = 9.33 mol/m^3^. The course of Curves 1A, 1B and 1 shows that for ${\bar{C}}_{1}\;$ < 9.15 mol/m^3^, $r_{12}^{B}$ > $r_{12}^{A}$ > $r_{12}$ and for ${\bar{C}}_{1}$ > 9.15 mol/m^3^, $r_{12}^{A}$ > $r_{12}^{B}$ >$r_{12}$. Similarly, Curves 2A, 2B and 2 show that for ${\bar{C}}_{1}\;$ < 9.33 mol/m^3^_,_ $r_{21}^{B}$ > $r_{21}^{A}$ > $r_{21}$ and for ${\bar{C}}_{1}\;$ > 9.33 mol/m^3^, $r_{21}^{A}$ > $r_{21}^{B}$ > $r_{21}$. Curves 1B and 2B have maxima. The coordinates of the maximum of Curve 1B are $r_{12}^{B}$ = 0.48 and ${\bar{C}}_{1}\;$ = 6.53 mol/m^3^. In turn, the coordinates of maximum of the 2B curve are $r_{21}^{B}$ = 0.46 and ${\bar{C}}_{1}\;$ = 7.14 mol/m^3^. This means that the maximum of Curve 1B is shifted relative to the maximum of Curve 2B vertically by ($r_{12}^{B}$ − $r_{21}^{B}$) = 0.02 and horizontally by $\Delta {\bar{C}}_{1}\;$ = 0.61 mol/m^3^. In addition, Curves 1A and 2A and Curves 1B and 2B are shifted relative to each other, except for the point with coordinates $r_{12}^{A}$ = $r_{21}^{A}$ = 0.14 and ${\bar{C}}_{1}\;$ = 2.15 mol/m^3^. This means that for ${\bar{C}}_{1}$ < 2.15 mol/m^3^ $r_{21}^{A}$ = $r_{12}^{A}$ while for ${\bar{C}}_{1}\;$ > 2.15 mol/m^3^ $r_{12}^{A}$ = $r_{21}^{A}$. Curves 1B and 2B coincide on the section with coordinates $r_{12}^{B}$ = $r_{21}^{B}$ = 0.48 and ${\bar{C}}_{1}$ = 8.03 mol/m^3^ and $r_{12}^{B}$ = $r_{21}^{B}$ = 0.33 and ${\bar{C}}_{1}$ = 9.47 mol/m^3^. For ${\bar{C}}_{1}$ < 8.03 mol/m^3^ and ${\bar{C}}_{1}$ > 9.47 mol/m^3^ the condition $r_{12}^{B}$ > $r_{21}^{B}$ is fulfilled. Curves 1 and 2 show that the condition $r_{12}$ = $r_{21}$ is fulfilled.

Figure 15 shows that Curves 1A and 1B intersect at a point with coordinates $r_{13}^{A}$ = $r_{13}^{B}$ = 0.17 and ${\bar{C}}_{1}$ = 9.1 mol/m^3^, and Curves 2A and 2B—at a point with coordinates $r_{31}^{A}$ = $r_{31}^{B}$ = 0.17 i ${\bar{C}}_{1}$ = 9.29 mol/m^3^. The course of Curves 1A, 1B and 1 shows that for ${\bar{C}}_{1}$ < 9.1 mol/m^3^, $r_{13}^{B}$ > $r_{13}^{A}$ > $r_{13}$ and for ${\bar{C}}_{1}$ > 9.1 mol/m^3^, $r_{13}^{A}$ > $r_{13}^{B}$ > $r_{13}$. Similarly, Curves 2A, 2B and 2 show that for ${\bar{C}}_{1}$ < 9.29 mol/m^3^, $r_{31}^{B}$ > $r_{31}^{A}$ > $r_{31}$ and for ${\bar{C}}_{1}$ > 9.29 mol/m^3^, $r_{31}^{A}$ > $r_{31}^{B}$ > $r_{31}$. Curves 1B and 2B overlap in the whole range of ${\bar{C}}_{1}$ used. Therefore, it can be assumed that $r_{13}^{B}$ = $r_{31}^{B}$. In turn, Curves 1A and 2A do not coincide only beyond the point with the coordinates: $r_{13}^{A}$ =$r_{31}^{A}$ = 0.24 and ${\bar{C}}_{1}$ = 11.25 mol/m^3^. For ${\bar{C}}_{1}$ > 11.25 mol/m^3^, $r_{13}^{A}$ > $r_{31}^{A}$. Curves 1 and 2 show that the condition $r_{12}$ = $r_{21}$ is fulfilled.

From the course of curves shown in Figure 16, it follows that $r_{23}^{A}$ = $r_{23}^{B}$ = $r_{23}$ and $r_{32}^{A}$ = $r_{32}^{B}$ = $r_{32}$. Curves 1 and 2 and 1A and 2B intersect at a point with coordinates $r_{23}$ = $r_{32}$ = $r_{23}^{A}$ = $r_{32}^{B}$ = 0.11 × 10^−3^ and ${\bar{C}}_{1}$ = 2.33 mol/m^3^, while Curves 1B and 2A—at a point with coordinates $r_{23}^{B}$ = $r_{32}^{A}$ = 0.12 and ${\bar{C}}_{1}$ = 2.6 mol/m^3^. For ${\bar{C}}_{1}$ < 2.33 mol/m^3^, $r_{23}$ = $r_{23}^{A}$ = $r_{23}^{B}$ > $r_{32}$ = $r_{32}^{A}$ = $r_{23}^{B}$ = $r_{32}^{B}$ and for ${\bar{C}}_{1}$ > 2.33 mol/m^3^, $r_{23}$ = $r_{23}^{A}$ = $r_{23}^{B}$ > $r_{32}$ = $r_{32}^{A}$ = $r_{32}^{B}$. As can be seen, the values of the coefficients $r_{ij}$, $r_{ij}^{r}$, $r_{ji}$ and $r_{ji}^{r}$ (*i*, *j* ∈ {2, 3} and *r* = A, B) (Figure 16) are three orders of magnitude smaller than the values of the coefficients $r_{ij}$, $r_{ij}^{r}$, $r_{ji}$ and $r_{ji}^{r}$ (*i*, *j* ∈ {1, 2} and *r* = A, B) and $r_{ij}$, $r_{ij}^{r}$, $r_{ji}$ and $r_{ji}^{r}$ (*i*, *j* ∈ {1, 3} and *r* = A, B) (Figure 14 and Figure 15).

Figure 14, Figure 15 and Figure 16 show that Kedem–Caplan relations take the form: 0.05 ≤ $r_{12}$ = $r_{21}$ ≤ 0.3, 0.1 ≤ $r_{12}^{A}$ ≤ 0.67, 0.15 ≤ $r_{12}^{B}$ ≤ 0.48, 0.11 ≤ $r_{21}^{A}$ ≤ 0.63, 0.15 ≤ $r_{21}^{B}$ ≤ 0.46, 0.1 ≤ $r_{13}$ = $r_{31}$ ≤ 0.11, 0.14 ≤ $r_{13}^{A}$ ≤ 0.24, 0.13 ≤ $r_{13}^{B}$ ≤ 0.28, 0.11 ≤ $r_{31}^{A}$ ≤ 0.24, 0.13 ≤ $r_{31}^{B}$ ≤ 0.25, 0.03 × 10^−3^ ≤ $r_{23}$ = $r_{23}^{A}$ = $r_{23}^{B}$ ≤ 0.18 × 10^−3^, 0.06 × 10^−3^ ≤ $r_{32}$ = $r_{32}^{A}$ = $r_{32}^{B}$ ≤ 0.36 × 10^−3^. Hence it follows that, $r_{12}^{A}$ ≠ $r_{21}^{A}$, $r_{12}^{B}$ ≠ $r_{21}^{B}$, $r_{13}^{A}$ ≈ $r_{31}^{A}$, $r_{13}^{B}$ = $r_{31}^{B}$, $r_{23}$ = $r_{23}^{A}$ = $r_{23}^{B}$ ≠ $r_{32}$ = $r_{32}^{A}$ = $r_{32}^{B}$. The values of all coupling coefficients presented in Figure 8a,b and Figure 9a fulfilled the conditions 0 ≤ $r_{ij}$ ≤ 1, 0 ≤ $r_{ij}^{r}$ ≤ 1, 0 ≤ $r_{ji}$ ≤ 1 and 0 ≤ $r_{ji}^{r}$ ≤ 1 determined by Roy Caplan [20].

Graphs in Figure 14 and Figure 15 have characteristic shapes, depending on the configuration of the membrane system and the properties of the solutions. In the case of homogeneous solutions (mechanically stirred solutions—Graphs 1 and 2), the coefficients do not depend on the configuration of the membrane system and are approximately linearly dependent on the concentration of glucose. This means that mechanical stirring of solutions at a sufficiently high speed eliminates CBL creation and causes maximization of fluxes and forces on the membrane. In the case of heterogeneous solutions (without mechanical stirring of the solutions in the chambers), the appearance of CBL near the membrane, reduces the value of the respective fluxes and increases the value of coupling factors for the same concentrations of solute in relation to homogeneous conditions. In addition, coupling coefficients for heterogeneous conditions strongly depend on the membrane configuration.

In Configuration A, the increase in glucose at a constant ethanol concentration at the beginning causes an increase in the coupling coefficients. In Configuration B, an increase in glucose causes a decrease in the value of coupling coefficients. The range of glucose concentrations for which the change in coupling coefficients in Configuration B is maximum is within the range similar to Configuration A of the membrane system. Analyzing the characteristics of coupling coefficients in heterogeneous conditions, we observed that for the respective characteristics in the A and B configurations of the membrane system, the respective graphs pairs (1A and 1B, 2A and 2B) intersect at a concentration of about 9.2 mol m^−3^. At this glucose concentration, the densities of the ternary solutions in the upper and lower chambers at the initial moment are the same. In this case, we observed the appearance of hydrodynamic instabilities that cause a disturbance of CBL diffusion reconstruction. Despite the fact that the solution densities were the same at the initial moment, the diffusion of glucose and ethanol through the membrane caused the appearance of sufficiently large and concentration gradients (and density gradients) in opposite direction to the gravitational field in the CBL areas. These gradients can cause hydrodynamic instabilities in the membrane system.

**Figure 17:**
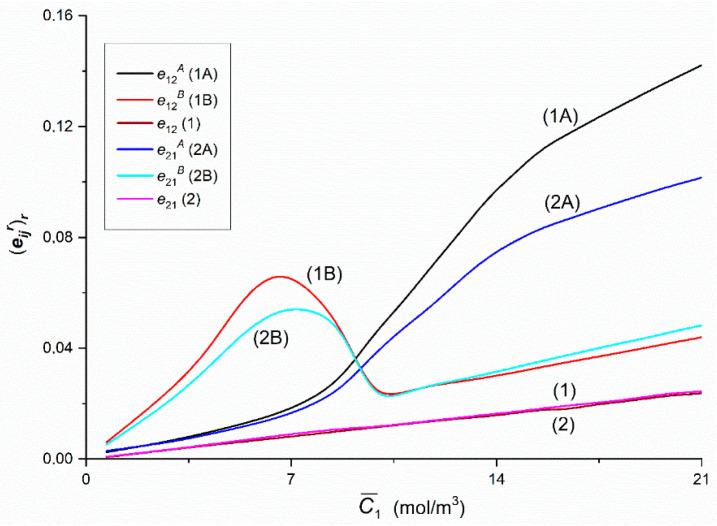
The ${\left(e_{ij}^{r}\right)}_{r}$ and ${\left(e_{ij}\right)}_{r}$ (*i*, *j* ∈ {1, 2}, *r* = A, B) coefficients as functions of glucose concentration.

Graphs in Figure 17 show that in the case of heterogeneous solutions (solutions not mechanically mixed—Graphs 1A and 1B, 2A and 2B), the coupling factors do not show their dependence on the configuration of the membrane system. Perhaps, because their value is very small.

**Figure 18:**
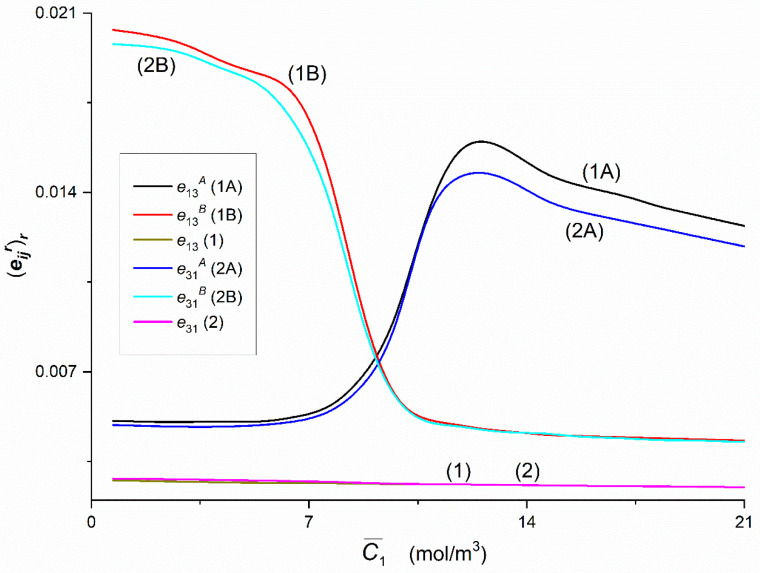
The ${\left(e_{ij}^{r}\right)}_{r}$ and ${\left(e_{ij}\right)}_{r}$ (*i*, *j* ∈ {1, 3}, *r* = A, B) coefficients as functions of glucose concentration.

**Figure 19:**
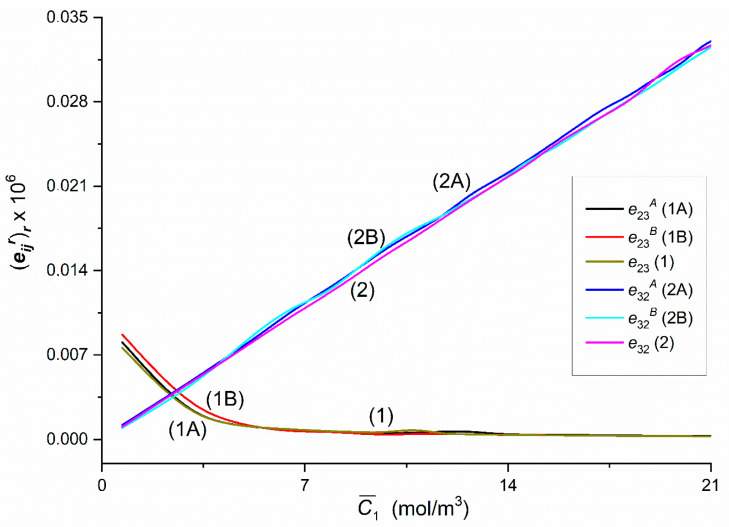
The ${\left(e_{ij}^{r}\right)}_{r}$ and ${\left(e_{ij}\right)}_{r}$ (*i*, *j* ∈ {2, 3} (10b), *r* = A, B) coefficients as functions of glucose concentration.

Figure 17, Figure 18 and Figure 19 show the dependences ${\left(e_{ij}^{r}\right)}_{r}$ = *f*(${\bar{C}}_{1}$, $\;{\bar{C}}_{2}$ = 37.71 mol/m^3^) and ${\left(e_{ij}\right)}_{r}$ = *f*(${\bar{C}}_{1}$, $\;{\bar{C}}_{2}$ = 37.71 mol/m^3^), (*i*, *j* ∈ {1, 2, 3} and *r* = A, B) calculated on the basis of Equations (13) and (14) and data presented in Figure 14, Figure 15 and Figure 16. Figure 17 shows that Curves 1A and 1B intersect at a point with coordinates:$\;{\left(e_{12}^{A}\right)}_{r}$ = ${\left(e_{12}^{B}\right)}_{r}$ = 0.036 and ${\bar{C}}_{1}$ = 9.18 mol/m^3^, and Curves 2A and 2B—at a point with coordinates:$\;{\left(e_{21}^{A}\right)}_{r}$ = ${\left(e_{21}^{B}\right)}_{r}$ = 0.032 and ${\bar{C}}_{1}$ = 9.41 mol/m^3^. The course of Curves 1A, 1B and 1 shows that for ${\bar{C}}_{1}$ < 9.18 mol/m^3^,$\;{\left(e_{12}^{B}\right)}_{r}$ > ${\left(e_{12}^{A}\right)}_{r}$ > ${\left(e_{12}\right)}_{r}$ and for ${\bar{C}}_{1}$ > 9.18 mol/m^3^,$\;{\left(e_{12}^{A}\right)}_{r}$ > ${\left(e_{12}^{B}\right)}_{r}$ > ${\left(e_{12}\right)}_{r}$. Similarly, the Curves 2A, 2B and 2 show that for ${\bar{C}}_{1}$ < 9.41 mol/m^3^_,_$\;{\left(e_{21}^{B}\right)}_{r}$ > ${\left(e_{21}^{A}\right)}_{r}$ > ${\left(e_{21}\right)}_{r}$ and for ${\bar{C}}_{1}$ > 9.41 mol/m^3^, ${\left(e_{21}^{A}\right)}_{r}$ > ${\left(e_{21}^{B}\right)}_{r}$ > ${\left(e_{21}\right)}_{r}$. Curves 1B and 2B have maxima. The coordinates of the maximum of Curve 1B are ${\left(e_{12}^{B}\right)}_{r}$ = 0.065 and ${\bar{C}}_{1}$ = 6.54 mol/m^3^. In turn, the coordinates of maximum of the 2B curve are ${\left(e_{21}^{B}\right)}_{r}$ = 0.054 and ${\bar{C}}_{1}$ = 7.23 mol/m^3^. This means that the maximum Curve 1B is shifted relative to the maximum Curve 2B vertically by ($r_{12}^{B}$ − $r_{21}^{B}$) = 0.011 and horizontally by $\Delta {\bar{C}}_{1}$ = 0.69 mol/m^3^. Curves 1B and 2B coincide on the section with coordinates: ${\left(e_{12}^{B}\right)}_{r}$ =${\left(e_{21}^{B}\right)}_{r}$ = 0.045 and ${\bar{C}}_{1}$ = 8.73 mol/m^3^ and ${\left(e_{12}^{B}\right)}_{r}$ = ${\left(e_{21}^{B}\right)}_{r}$ = 0.028 and ${\bar{C}}_{1}$ = 13.09 mol/m^3^. For ${\bar{C}}_{1}$ < 8.73 mol/m^3^ the conditions: ${\left(e_{12}^{B}\right)}_{r}$ > ${\left(e_{21}^{B}\right)}_{r}$ (for ${\bar{C}}_{1}$ > 13.09 mol/m^3^) and ${\left(e_{12}^{B}\right)}_{r}$ < ${\left(e_{21}^{B}\right)}_{r}$ are fulfilled. Curves 1 and 2 show that the condition ${\left(e_{12}\right)}_{r}$ = ${\left(e_{21}\right)}_{r}$ is fulfilled.

Figure 18 shows that Graphs 1A, 1B, 2A and 2B intersect approximately at the point with the coordinates ${\left(e_{13}^{A}\right)}_{r}$ = ${\left(e_{13}^{B}\right)}_{r}$ ≈ 0.008 and ${\bar{C}}_{1}$ = 9.24 mol/m^3^. Curves 1A, 1B and 1 show that for ${\bar{C}}_{1}$ < 9.24 mol/m^3^ ${\left(e_{13}^{B}\right)}_{r}$ > ${\left(e_{13}^{A}\right)}_{r}$ > ${\left(e_{13}\right)}_{r}$ and for ${\bar{C}}_{1}$ > 9.24 mol/m^3^ ${\left(e_{13}^{A}\right)}_{r}$ > ${\left(e_{13}^{B}\right)}_{r}$ > ${\left(e_{13}\right)}_{r}$. Similarly, the course of Curves 2A, 2B and 2 shows that for ${\bar{C}}_{1}$ < 9.24 mol/m^3^ ${\left(e_{31}^{B}\right)}_{r}$ > ${\left(e_{31}^{A}\right)}_{r}$ > ${\left(e_{31}\right)}_{r}$ and for ${\bar{C}}_{1}$ > 9.24 mol/m^3^ ${\left(e_{31}^{A}\right)}_{r}$ > ${\left(e_{31}^{B}\right)}_{r}$ > ${\left(e_{31}\right)}_{r}$. Curves 1B and 2B coincide for ${\bar{C}}_{1}$ > 9.24 mol/m^3^. Therefore, it can be assumed that for this concentration range ${\left(e_{31}^{A}\right)}_{r}$ > ${\left(e_{31}^{B}\right)}_{r}$ > ${\left(e_{31}\right)}_{r}$. In the other ranges ${\bar{C}}_{1}$ Curves 1A and 2A do not cover. This means that ${\left(e_{13}^{B}\right)}_{r}$ > ${\left(e_{31}^{B}\right)}_{r}$ and ${\left(e_{13}^{A}\right)}_{r}$ > ${\left(e_{31}^{A}\right)}_{r}$. Curves 1 and 2 show that the condition ${\left(e_{13}\right)}_{r}$ = ${\left(e_{31}\right)}_{r}$.

From the course of Curves 1A, 1B and 1 presented in Figure 19 it follows that ${\left(e_{23}^{A}\right)}_{r}$ = ${\left(e_{23}^{B}\right)}_{r}$ = ${\left(e_{23}\right)}_{r}$ and ${\left(e_{32}^{A}\right)}_{r}$ = ${\left(e_{32}^{B}\right)}_{r}$ = ${\left(e_{32}\right)}_{r}$. Curves 1, 1A and 1B and 2, 2A and 2B intersect at a point with coordinates ${\left(e_{23}\right)}_{r}$ = ${\left(e_{23}^{A}\right)}_{r}$ = ${\left(e_{23}^{B}\right)}_{r}$ = ${\left(e_{32}\right)}_{r}$ = ${\left(e_{32}^{A}\right)}_{r}$ = ${\left(e_{32}^{B}\right)}_{r}$ ≈ 0.004 and ${\bar{C}}_{1}$ = 2.57 mol/m^3^. For ${\bar{C}}_{1}$ < 2.57 mol/m^3^, ${\left(e_{23}\right)}_{r}$ = ${\left(e_{23}^{A}\right)}_{r}$ = ${\left(e_{23}^{B}\right)}_{r}$ > ${\left(e_{32}\right)}_{r}$ = ${\left(e_{32}^{A}\right)}_{r}$ = ${\left(e_{32}^{B}\right)}_{r}$ and for ${\bar{C}}_{1}$ > 2.57 mol/m^3^, ${\left(e_{23}\right)}_{r}$ = ${\left(e_{23}^{A}\right)}_{r}$ = ${\left(e_{23}^{B}\right)}_{r}$ < ${\left(e_{32}\right)}_{r}$ = ${\left(e_{32}^{A}\right)}_{r}$ = ${\left(e_{32}^{B}\right)}_{r}$.

Figure 17, Figure 18 and Figure 19 show that Kedem–Caplan relations take the form: 0.005 ≤ ${\left(e_{12}\right)}_{r}$ = ${\left(e_{21}\right)}_{r}$ ≤ 0.05, 0.002 ≤ ${\left(e_{12}^{A}\right)}_{r}$ ≤ 0.145, 0.006 ≤ ${\left(e_{12}^{B}\right)}_{r}$ ≤ 0.068, 0.003 ≤ ${\left(e_{21}^{A}\right)}_{r}$ ≤ 0.104, 0.005 ≤ ${\left(e_{21}^{B}\right)}_{r}$ ≤ 0.054, 0.004 ≤ ${\left(e_{13}\right)}_{r}$ = ${\left(e_{31}\right)}_{r}$ ≤ 0.02, 0.005 ≤ ${\left(e_{13}^{A}\right)}_{r}$ ≤ 0.016, 0.004 ≤ ${\left(e_{13}^{B}\right)}_{r}$ ≤ 0.02, 0.005 ≤ ${\left(e_{31}^{A}\right)}_{r}$ ≤ 0.015, 0.04 ≤ ${\left(e_{31}^{B}\right)}_{r}$ ≤ 0.02, 0.003 × 10^−6^ ≤ ${\left(e_{23}\right)}_{r}$ = ${\left(e_{23}^{A}\right)}_{r}$ = ${\left(e_{23}^{B}\right)}_{r}$ ≤ 0.009 × 10^−6^, 0.001 × 10^−6^ ≤ ${\left(e_{32}\right)}_{r}$ = ${\left(e_{32}^{A}\right)}_{r}$ = ${\left(e_{32}^{B}\right)}_{r}$ ≤ 0.034 × 10^−6^. Hence it follows that, ${\left(e_{12}^{A}\right)}_{r}$ ≠ ${\left(e_{21}^{A}\right)}_{r}$, ${\left(e_{12}^{B}\right)}_{r}$ ≠ ${\left(e_{21}^{B}\right)}_{r}$, ${\left(e_{13}^{A}\right)}_{r}$ ≈ ${\left(e_{31}^{A}\right)}_{r}$, ${\left(e_{13}^{B}\right)}_{r}$ = ${\left(e_{31}^{B}\right)}_{r}$, ${\left(e_{23}\right)}_{r}$ = ${\left(e_{23}^{A}\right)}_{r}$ = ${\left(e_{23}^{B}\right)}_{r}$ ≠ ${\left(e_{32}\right)}_{r}$ = ${\left(e_{32}^{A}\right)}_{r}$ = ${\left(e_{32}^{B}\right)}_{r}$. The values of all coupling coefficients presented in Figure 14, Figure 15 and Figure 16 fulfill the conditions 0 ≤ ${\left(e_{ij}\right)}_{r}$ ≤ 1, 0 ≤ ${\left(e_{ij}^{A}\right)}_{r}$ ≤ 1, 0 ≤ ${\left(e_{ji}\right)}_{r}$ ≤ 1, 0 ≤ ${\left(e_{ji}^{A}\right)}_{r}$ ≤ 1 determined by Roy Caplan [20].

**Figure 20:**
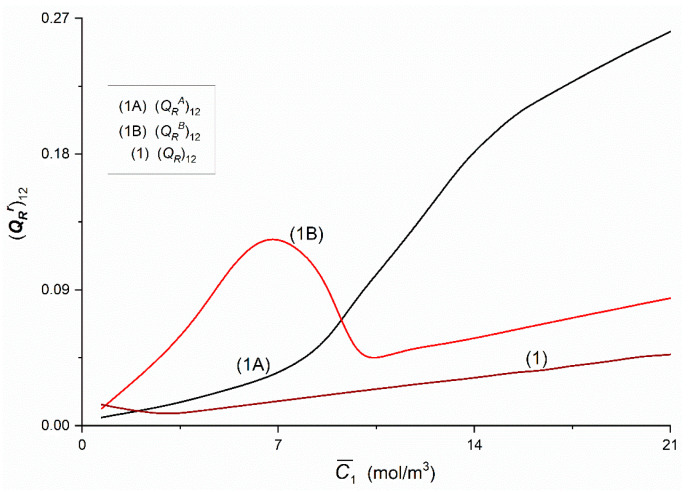
The ${\left(Q_{R}^{r}\right)}_{ij}$ and ${\left(Q_{R}\right)}_{ij}$ (*i*, *j* ∈ {1, 2}, *r* = A, B) coefficients as functions of glucose concentration.

**Figure 21:**
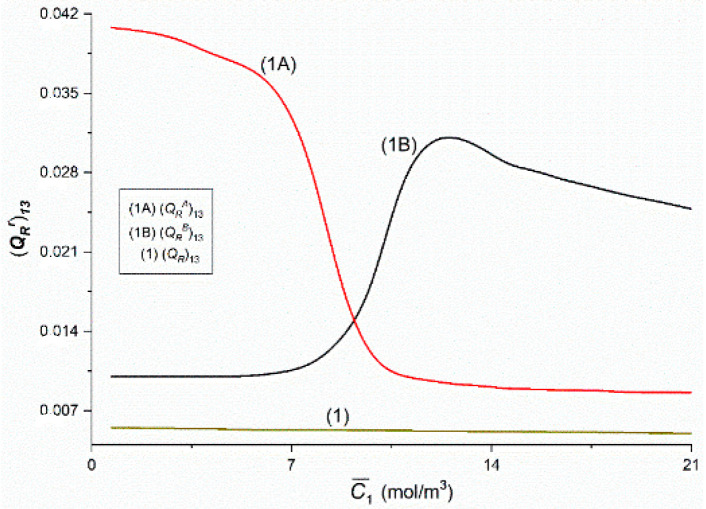
The ${\left(Q_{R}^{r}\right)}_{ij}$ and ${\left(Q_{R}\right)}_{ij}$ (*i*, *j* ∈ {1, 3}, *r* = A, B) coefficients as functions of glucose concentration.

Figure 20 and Figure 21 show the dependences ${\left(Q_{R}^{r}\right)}_{ij}$ = *f*(${\bar{C}}_{1}$, $\;{\bar{C}}_{2}$ = 37.71 mol/m^3^) and ${\left(Q_{r}\right)}_{ij}$ = *f*(${\bar{C}}_{1}$, $\;{\bar{C}}_{2}$ = 37.71 mol/m^3^), (*i*, *j* ∈ {1, 2, 3} and *r* = A, B) calculated on the basis of Equations (15) and (16) and data presented in Figure 14, Figure 15 and Figure 16. Figure 20 shows that Curves 1A and 1B intersect at a point with coordinates:$\;{\left(Q_{R}^{A}\right)}_{12}$ = ${\left(Q_{R}^{B}\right)}_{12}$ = 0.07 and ${\bar{C}}_{1}$ = 9.24 mol/m^3^. The course of Curves 1A, 1B and 1 shows that for ${\bar{C}}_{1}$ < 9.24 mol/m^3^,$\;{\left(Q_{R}^{B}\right)}_{12}$ > ${\left(Q_{R}^{A}\right)}_{12}$ > ${\left(Q_{R}\right)}_{12}$ and for ${\bar{C}}_{1}$ > 9.24 mol/m^3^, ${\left(Q_{R}^{A}\right)}_{12}$ > ${\left(Q_{R}^{B}\right)}_{12}$ > ${\left(Q_{R}\right)}_{12}$. Curve 1B has a maximum. The coordinates of the maximum of Curve 1B are ${\left(Q_{R}^{B}\right)}_{12}$ = 0.12 and ${\bar{C}}_{1}$ = 6.77 mol/m^3^. Figure 21 shows that Graphs 1A and 1B intersect at the point with the coordinates ${\left(Q_{R}^{A}\right)}_{13}$ = ${\left(Q_{R}^{B}\right)}_{13}$ = 0.015 and ${\bar{C}}_{1}$ = 9.16 mol/m^3^. Curves 1A, 1B and 1 show that for ${\bar{C}}_{1}$ < 9.24 mol/m^3^ ${\left(Q_{R}^{B}\right)}_{13}$ > ${\left(Q_{R}^{A}\right)}_{13}$ > ${\left(Q_{R}\right)}_{13}$ and for ${\bar{C}}_{1}$ > 9.24 mol/m^3^ ${\left(Q_{R}^{A}\right)}_{13}$ > ${\left(Q_{R}^{B}\right)}_{13}$ > ${\left(Q_{R}\right)}_{13}$. Moreover, it was shown that ${\left(Q_{R}^{B}\right)}_{23}$ = ${\left(Q_{R}^{A}\right)}_{23}$ = ${\left(Q_{R}\right)}_{23}$ = 0.58 × 10^−8^ = constant. Figure 20 and Figure 21 show that Kedem–Caplan relations take the form: 0.01 ≤ ${\left(Q_{R}\right)}_{12}$ ≤ 0.05, 0.05 ≤ ${\left(Q_{R}^{A}\right)}_{12}$ ≤ 0.27, 0.01 ≤ ${\left(Q_{R}^{B}\right)}_{12}$ ≤ 0.13, 0.005 ≤ ${\left(Q_{R}\right)}_{13}$ ≤ 0.0055, 0.008 ≤ ${\left(Q_{R}^{A}\right)}_{13}$ ≤ 0.041, 0.01 ≤ ${\left(Q_{R}^{B}\right)}_{13}$ ≤ 0.031. The values of all coupling coefficients presented in Figure 20 and Figure 21 fulfill the conditions 0 ≤ ${\left(Q_{R}\right)}_{ij}$ ≤ 1 and 0 ≤ ${\left(Q_{R}^{r}\right)}_{ij}$ ≤ 1.

The results of experimental research indicate that *ω*_11_ >> *ω*_12_, *ω*_22_ >> *ω*_21_, $\zeta_{p}^{r}$ = $\zeta_{a1}^{r}$ = $\zeta_{a2}^{r}$ = =1, $\zeta_{v1}^{r}$ = $\zeta_{s11}^{r}$ = $\zeta_{s12}^{r}$ = $\zeta_{1}^{r}$ and $\zeta_{v2}^{r}$ = $\zeta_{s22}^{r}$ = $\zeta_{s21}^{r}$ = $\zeta_{2}^{r}$ (r = A, B). By accepting the above conditions and that $\zeta_{1}^{r}$ ≈ $\zeta_{2}^{r}$ = $\zeta^{r}$. Given this condition, and Equations (5), (9) and (10), we can write:

(17) $$\xi_{11}=\frac{\zeta^{A}-\zeta^{B}}{\zeta^{A}\zeta^{B}}\frac{L_{p}[{\bar{C}}_{1}\omega_{22}\left(1-\sigma_{1}\right)+{\bar{C}}_{2}\omega_{11}\left(1-\sigma_{2}\right)]}{\omega_{11}\omega_{22}+L_{p}\left[\omega_{22}{\bar{C}}_{1}{\left(1-\sigma_{1}\right)}^{2}+\omega_{11}{\bar{C}}_{2}{\left(1-\sigma_{2}\right)}^{2}\right]}$$

(18) $$\xi_{12}=\frac{\zeta^{A}-\zeta^{B}}{\zeta^{A}\zeta^{B}}\frac{1}{\left(1-\sigma_{1}\right)}$$

(19) $$\xi_{13}=\frac{\zeta^{A}-\zeta^{B}}{\zeta^{A}\zeta^{B}}\frac{1}{\left(1-\sigma_{2}\right)}$$

(20) $$\xi_{21}=-\frac{\zeta^{A}-\zeta^{B}}{\zeta^{A}\zeta^{B}}=\xi_{31}=-\xi_{22}=-\xi_{23}=-\xi_{32}=-\xi_{33}$$

(21) $$\xi_{det}=\frac{{\left(\zeta^{A}\right)}^{2}-{\left(\zeta^{B}\right)}^{2}}{{\left(\zeta^{A}\right)}^{2}{\left(\zeta^{B}\right)}^{2}}$$

Equations (17)–(20) contain the factor $\left(\zeta_{1}^{A}-\zeta_{1}^{B}\right){\left(\zeta_{1}^{A}\zeta_{1}^{B}\right)}^{-1}$ and Equation (22)—the factor $\left[{\left(\zeta^{A}\right)}^{2}-{\left(\zeta^{B}\right)}^{2}\right]{\left[{\left(\zeta^{A}\right)}^{2}{\left(\zeta^{B}\right)}^{2}\right]}^{-1}$. This factor, using Equation (1) can be written in a form containing the thickness of CBLs. To simplify the accounts, using the conditions ${\left(D_{ij}^{r}\right)}_{l}$ = ${\left(D_{ij}^{r}\right)}_{h}$ = *D_ij_* and $\delta_{h}^{r}$ = $\delta_{l}^{r}$ = *δ^r^*, we write the Equation (1) in the form:

(22) $$\zeta^{r}=\frac{D_{ij}}{D_{ij}+2RT\omega_{ij}\delta^{r}}$$

Using Equation (22) we can write:

(23) $$\frac{\zeta^{A}-\zeta^{B}}{\zeta^{A}\zeta^{B}}=\frac{2RT\omega_{ij}\left(\delta^{B}-\delta^{A}\right)}{D_{ij}}\;$$

(24) $$\frac{{\left(\zeta^{A}\right)}^{2}-{\left(\zeta^{B}\right)}^{2}}{{\left(\zeta^{A}\right)}^{2}{\left(\zeta^{B}\right)}^{2}}\;=\;\frac{4RT\omega_{ij}}{D_{{ij}^{2}}}\;\{D_{ij}\left(\delta^{B}-\delta^{A}\right)\;+\;RT\omega_{ij}\left[{\left(\delta^{B}\right)}^{2}\;-\;{\left(\delta^{A}\right)}^{2}\right]\}$$

From all the foregoing considerations, it is clear that coefficients $\xi_{ij}$ (*i*, *j* ∈ {1, 2, 3} and $\xi_{det}$ are measures of the natural convection effect. If the conditions $\xi_{ij}$ < 0 and $\xi_{det}$ < 0 are fulfilled, fluxes of natural convection in single-membrane system are directed vertically upwards. In turn, for coefficients $\xi_{ij}$ > 0 and $\xi_{det}$ > 0, the fluxes are directed vertically downwards. Zeroing of the coefficients ($\xi_{ij}$ = 0 and $\xi_{det}$ = 0) means that the system is in the critical point where the flux turns its direction from vertically upwards to vertically downwards. In this point, the structure of layers lose its stability, but natural convection does not have precise turn yet, what means that the membrane system is not sensitive to changes in the gravitational field. This is shown by dependencies $\xi_{ij}$ = *f*(${\bar{C}}_{1}$, $\;{\bar{C}}_{2}\;$ = 37.71 mol/m^3^), (*i*, *j* ∈ {1, 2, 3} and $\xi_{det}$ = *f*(${\bar{C}}_{1}$, $\;{\bar{C}}_{2}\;$ = 37.71 mol/m^3^), presented in Figure 11, Figure 12 and Figure 13 as well as interferograms presented in the previous publication [37,38]. Hydrodynamic stability in the membrane system is controlled by the concentration Rayleigh number [34,35,36,37,38]. The Rayleigh number value depends on the concentration of solutions separated by the membrane [34,35]. For the points where $\xi_{ij}$ = 0 and $\xi_{det}$ = 0, (*i*, *j* ∈ {1, 2, 3}) the critical value of concentration Rayleigh number (*R_C_*) can be specified.

For example, we will consider Equations (20) and (23) and Figure 2 and Figure 3 for the $\xi_{22}$ coefficient. This equation can be written as $\xi_{22}=2RT\omega_{11}\left(\delta^{B}-\delta^{A}\right)D_{11}{}^{-1}$. It is drawn from the equation and Figure 2 that if $\xi_{22}$ = 0, then $\zeta_{1}^{A}$ = $\zeta_{1}^{B}$ = 0.234. From the equation, it becomes apparent that if $\xi_{22}$ = 0, then $\delta^{A}$ = $\delta^{B}$. The values of $\delta^{A}$ and $\delta^{B}$ can be determined by laser interferometry [35,36,37,38] or volume flux measurements [34]. Figure 3 presents the dependences $\delta^{r}$ = *f*(*ρ_h_* − *ρ_l_*) obtained by converting the dependence $\zeta_{i}^{r}$ = *f*(${\bar{C}}_{1},\;{\bar{C}}_{2}\;$ = const.) shown in Figure 3, with the help of equations $\delta^{r}=D_{ij}\left(1-\zeta_{i}^{r}\right){\left(2RT\omega_{ij}\zeta_{i}^{r}\right)}^{-1}$ and *ρ_h_* − *ρ_l_* = (∂*ρ*/∂*C*_1_)(*C*_1*h*_ − *C*_1*l*_) + (∂*ρ*/∂*C*_2_)(*C*_2*h*_ − *C*_2*l*_). From this figure it follows that $\delta^{A}$ = $\delta^{B}$ ≈ 1.3 × 10^−3^ m for *ρ_h_* − *ρ_l_* = 0.046 kg/m^3^.

Let us consider the dependency $\xi_{22}$ = *f*(${\bar{C}}_{1}$, $\;{\bar{C}}_{2}$ = 37.71 mol/m^3^) shown in the Figure 12. It results from the figure that $\xi_{22}$ = 0 for ${\bar{C}}_{1}$ = 9.24 mol/m^3^ and ${\bar{C}}_{2}$ = 37.71 mol/m^3^. It should be pointed out that ${\bar{C}}_{1}$ = 9.24 mol/m^3^ if *C*_1*h*_ = 33.44 mol/m^3^ and *C*_1*l*_ = 1 mol m^−3^ while ${\bar{C}}_{2}$ = 37.71 mol/m^3^, for *C*_2*h*_ = 201 mol/m^3^ and *C*_2*l*_ = 1 mol/m^3^. Therefore, consisting solution density amounts to 998.3 kg/m^3^. In turn, kinematic viscosity of this solution is equal to *ν* = 1.063 × 10^−6^ m^2^/s. Density difference of solutions located in the Compartments (h) and (l) calculated on the basis of equation *ρ_h_* − *ρ_l_* = (∂*ρ*/∂*C*_1_)(*C*_1*h*_ − *C*_1*l*_) + (∂*ρ*/∂*C*_2_)(*C*_2*h*_ − *C*_2*l*_), where (∂*ρ*/∂*C*_1_) = 0.06 kg/mol, (∂*ρ*/∂*C*_2_) = −0.0095 kg/mol, amounts to *ρ_h_* − *ρ_l_* = 0.046 kg/m^3^. Taking these data into consideration, as well as *D*_11_ = 0.69 × 10^−9^ m^2^/s, *g* = 9.81 m/s^2^, *ω*_11_ = 0.8 × 10^−9^ mol/Ns, δ = 1.3 × 10^−3^ m in the expression for the concentration Rayleigh number *R_C_* = [*g*(*ρ_h_* − *ρ_l_*)(*δ*)^3^](*ρ_h_ν_h_D*_11_)^−1^ [29,30], we get *R_C_* = 1353.1. This value corresponds to the (*R_C_*)_crit._ = 1100.6, obtained for the case of the rigid membrane surface and the free liquid interior (rigid-free borders) [44,45]. For electrolysis occurring in a cell containing electrodes placed in parallel in horizontal planes, the critical Rayleigh number depends strongly on the distance between these electrodes and for amperostatic conditions takes the values in the range of *R_C_* = 1070 ÷ 1540 [46]. In turn, for potentiostatic conditions *R_C_* takes the values in the range of *R_C_* = 763.3 ÷ 1351 [47].

## 4. Conclusions

From the above presented studies, the following results are obtained:

1. In order to describe transport processes of ternary solutions of nonelectrolytes through horizontally oriented membrane, nine Peusner’s coefficients should be calculated $R_{ij}^{r}$ (*i*, *j* ∈ {1, 2, 3}, *r* = A, B) and the determinant of the matrix of these coefficients is *det* [*R^r^*] = $R_{det}^{r}$. For the Nephrophan membrane and aqueous solutions of glucose and ethanol, the values of coefficients $R_{ij}^{r}$ (*i*, *j* ∈ {1, 2, 3}, *r* = A, B) and $R_{det}^{r}$ are dependent on concentration solutions and configuration of the membrane system. For *i* ≠ *j* these coefficients fulfill the relations $R_{ij}^{r}$ ≠ $R_{ji}^{r}$.
2. Concentration dependencies of coefficients $\xi_{ij}$ = ($R_{ij}^{A}$ − $R_{ij}^{B}$)/*R_ij_* = *f*(${\bar{C}}_{1}$, $\;{\bar{C}}_{2}$ = 37.71 mol/m^3^) and $\xi_{det}$ = (*det* [*R^A^*] – *det* [*R^B^*])/*det* [*R*]) = *f*(${\bar{C}}_{1}$, $\;{\bar{C}}_{2}$ = 37.71 mol/m^3^) facilitate estimation of natural convection direction: for $\xi_{ij}$ < 0, natural convection is directed vertically upwards and for $\xi_{ij}$ > 0—vertically downwards. The value of coefficients $\xi_{ij}$ and $\xi_{det}$ ($\xi_{ij}$ < 0, $\xi_{det}$ < 0, $\xi_{ij}$ = 0, $\xi_{det}$ = 0, $\xi_{ij}$ > 0 or $\xi_{det}$ > 0) shows the influence of concentration polarization and natural convection on the membrane transport. For $\xi_{ij}$ = 0 the critical value of the concentration Rayleigh number (*R_C_*) can be estimated, for the point where convective stream changes its direction from vertical upwards into vertical downwards. The *R_C_* value estimated in this paper for the considered case amounts to (*R_C_*)_crit._ = 1353.1.
3. For (*i*, *j* ∈ {1, 2}, *r* = A, B) the coupling ($r_{ij}^{r}$), ${\left(Q_{R}^{r}\right)}_{ij}$ and energy conversion ${\left(e_{ij}^{r}\right)}_{r}$ Coefficients depend on the concentration of homogeneous solutions and in concentration polarization conditions—on the concentration of solutions and the configuration of the membrane system. For (*i*, *j* ∈ {1, 3}, *r* = A, B) these coefficients in concentration polarization conditions depend and in homogeneous solutions do not depend on the concentration of solutions and the configuration of the membrane system. The crisscrosses of suitable A and B characteristics are observed at a glucose concentration ${\bar{C}}_{1}$ = 9.24 mol m^−3^. For (*i*, *j* ∈ {2, 3}, *r* = A, B) the coefficients ($r_{ij}^{r}$) and ${\left(e_{ij}^{r}\right)}_{r}$ depend on the concentration of homogeneous solutions and in concentration polarization conditions and do not depend on the configuration of the membrane system. The crisscrosses of suitable A and B characteristics are observed at a glucose concentration ${\bar{C}}_{1}$ ≈ 2.5 mol m^−3^. The ${\left(Q_{R}^{r}\right)}_{ij}$ coefficient is independent of the concentration and configuration of the membrane system.
4. Curves marked with a number and the letters A or B are evidence that there are transition points associated with the change in the nature of membrane transport from osmotic-diffusion to osmotic-diffusion-convective or vice versa. This means that in Configuration A, we have a transition from convective to convective, and in Configuration B—from convective to non-convective. These transitions are a pseudo-phase transition.
5. The presented equations are a new research tool for membrane transport and the influence of gravity field on this transport.

## Figures and Tables

**Table 1:** Criteria for coefficients, $R_{ij}^{A}$, $R_{ij}^{B}$, $R_{ij}$, $R_{det}^{A}$, $R_{det}^{B}$, $\xi_{ij}$ and $\xi_{det}$.

$R_{ij}^{A}$ > 0, $R_{ij}^{B}$ > 0, *R_ij_* > 0	$R_{ij}^{A}$ > *R_ij_*, $R_{ij}^{B}$ > *R_ij_* $R_{ij}^{A}$ < *R_ij_*, $R_{ij}^{B}$ < *R_ij_*	$R_{ij}^{A}$ > $R_{ij}^{B}$ $R_{ij}^{A}$ < $R_{ij}^{B}$ $R_{ij}^{A}$ = $R_{ij}^{B}$	*ξ_ij_* > 0 *ξ_ij_* < 0 *ξ_ij_* = 0
$R_{ij}^{A}$ < 0, $R_{ij}^{B}$ < 0, *R_ij_* < 0	$R_{ij}^{A}$ > *R_ij_*, $R_{ij}^{B}$ > *R_ij_* $R_{ij}^{A}$ < *R_ij_*, $R_{ij}^{B}$ < *R_ij_*	$R_{ij}^{A}$ > $R_{ij}^{B}$ $R_{ij}^{A}$ < $R_{ij}^{B}$ $R_{ij}^{A}$ = $R_{ij}^{B}$	*ξ_ij_* < 0 *ξ_ij_* > 0 *ξ_ij_* = 0
$R_{det}^{A}$ > 0, $R_{det}^{B}$ > 0, *R_det_* > 0	$R_{det}^{A}$ > *R_det_*, $R_{det}^{B}$ > *R_det_* $R_{det}^{A}$ < *R_det_*, $R_{det}^{B}$ < *R_det_*	$R_{det}^{A}$ > $R_{det}^{B}$ $R_{det}^{A}$ < $R_{det}^{B}$ $R_{det}^{A}$ = $R_{det}^{B}$	*ξ_det_* > 0 *ξ_det_* < 0 *ξ_det_* = 0
$R_{det}^{A}$ < 0, $R_{det}^{B}$ < 0, *R_det_* < 0	$R_{det}^{A}$ > *R_det_*, $R_{det}^{B}$ > *R_det_* $R_{det}^{A}$ < *R_det_*, $R_{det}^{B}$ < *R_det_*	$R_{det}^{A}$ > $R_{det}^{B}$ $R_{det}^{A}$ < $R_{det}^{B}$ $R_{det}^{A}$ = $R_{det}^{B}$	*ξ_det_* < 0 *ξ_det_* > 0 *ξ_det_* = 0

**Table 2:** Relationships between coefficients $R_{ij}^{A}$, $R_{ij}^{B}$, $R_{ij}$, $\xi_{ij}$ (i, j ∈ {1, 2, 3}, $R_{det}^{A}$, $R_{det}^{B}$ and $\xi_{det}$.

$R_{11}^{A}$ > 0, $R_{11}^{B}$ > 0, $R_{11}$ > 0	$R_{11}^{A}$ < $R_{11}^{B}$, $R_{11}$ < $R_{11}^{A}$, $R_{11}$ < $R_{11}^{B}$	$\xi_{11}$ < 0
	$R_{11}^{A}$ > $R_{11}^{B}$, $R_{11}$ < $R_{11}^{A}$, $R_{11}$ < $R_{11}^{B}$	$\xi_{11}$ > 0
	$R_{11}^{A}$ = $R_{11}^{B}$, $R_{11}$ < $R_{11}^{A}$, $R_{11}$ < $R_{11}^{B}$	$\xi_{11}$ = 0
$R_{12}^{A}$ < 0, $R_{12}^{B}$ < 0, $R_{12}$ < 0	$R_{12}^{A}$ > $R_{12}^{B}$, $R_{12}$ > $R_{12}^{A}$, $R_{12}$ > $R_{12}^{B}$	$\xi_{12}$ < 0
	$R_{12}^{A}$ < $R_{12}^{B}$, $R_{12}$ > $R_{12}^{A}$, $R_{12}$ > $R_{12}^{B}$	$\xi_{12}$ > 0
	$R_{12}^{A}$ = $R_{12}^{B}$, $R_{12}$ > $R_{12}^{A}$, $R_{12}$ > $R_{12}^{B}$	$\xi_{12}$ = 0
$R_{21}^{A}$ < 0, $R_{21}^{B}$ < 0, $R_{21}$ < 0	$R_{21}^{A}$ > $R_{21}^{B}$, $R_{21}$ > $R_{21}^{A}$, $R_{21}$ > $R_{21}^{B}$	$\xi_{21}$ < 0
	$R_{21}^{A}$ < $R_{21}^{B}$, $R_{21}$ > $R_{21}^{A}$, $R_{21}$ > $R_{21}^{B}$	$\xi_{21}$ > 0
	$R_{21}^{A}$ = $R_{21}^{B}$, $R_{21}$ > $R_{21}^{A}$, $R_{21}$ > $R_{21}^{B}$	$\xi_{21}$ = 0
$R_{13}^{A}$ < 0, $R_{13}^{B}$ < 0, $R_{13}$ < 0	$R_{13}^{A}$ > $R_{13}^{B}$, $R_{13}$ > $R_{13}^{A}$, $R_{13}$ > $R_{13}^{B}$	$\xi_{13}$ < 0
	$R_{13}^{A}$ < $R_{13}^{B}$, $R_{13}$ > $R_{13}^{A}$, $R_{13}$ > $R_{13}^{B}$	$\xi_{13}$ > 0
	$R_{13}^{A}$ = $R_{13}^{B}$, $R_{13}$ > $R_{13}^{A}$, $R_{13}$ > $R_{13}^{B}$	$\xi_{13}$ = 0
$R_{31}^{A}$ < 0, $R_{31}^{B}$ < 0, $R_{31}$ < 0	$R_{31}^{A}$ > $R_{31}^{B}$, $R_{31}$ > $R_{31}^{A}$, $R_{31}$ > $R_{31}^{B}$	$\xi_{31}$ < 0
	$R_{31}^{A}$ < $R_{31}^{B}$, $R_{31}$ > $R_{31}^{A}$, $R_{31}$ > $R_{31}^{B}$	$\xi_{31}$ > 0
	$R_{31}^{A}$ = $R_{31}^{B}$, $R_{31}$ > $R_{31}^{A}$, $R_{31}$ > $R_{31}^{B}$	$\xi_{31}$ = 0
$R_{22}^{A}$ > 0, $R_{22}^{B}$ > 0, $R_{22}$ > 0	$R_{22}^{A}$ < $R_{22}^{B}$, $R_{22}$ < $R_{22}^{A}$, $R_{22}$ < $R_{22}^{B}$	$\xi_{22}$ < 0
	$R_{22}^{A}$ > $R_{22}^{B}$, $R_{22}$ < $R_{22}^{A}$, $R_{22}$ < $R_{22}^{B}$	$\xi_{22}$ > 0
	$R_{22}^{A}$ = $R_{22}^{B}$, $R_{22}$ < $R_{22}^{A}$, $R_{22}$ < $R_{22}^{B}$	$\xi_{22}$ = 0
$R_{23}^{A}$ < 0, $R_{23}^{B}$ < 0, $R_{23}$ < 0	$R_{23}^{A}$ > $R_{23}^{B}$, $R_{23}$ > $R_{23}^{A}$, $R_{23}$ > $R_{23}^{B}$	$\xi_{23}$ < 0
	$R_{23}^{A}$ < $R_{23}^{B}$, $R_{23}$ > $R_{23}^{A}$, $R_{23}$ > $R_{23}^{B}$	$\xi_{23}$ > 0
	$R_{23}^{A}$ = $R_{23}^{B}$, $R_{23}$ > $R_{23}^{A}$, $R_{23}$ > $R_{23}^{B}$	$\xi_{23}$ = 0
$R_{32}^{A}$ < 0, $R_{32}^{B}$ < 0, $R_{32}$ < 0	$R_{32}^{A}$ > $R_{32}^{B}$, $R_{32}$ > $R_{32}^{A}$, $R_{32}$ > $R_{23}^{B}$	$\xi_{32}$ < 0
	$R_{32}^{A}$ < $R_{32}^{B}$, $R_{32}$ > $R_{32}^{A}$, $R_{32}$ > $R_{23}^{B}$	$\xi_{32}$ > 0
	$R_{32}^{A}$ = $R_{32}^{B}$, $R_{32}$ > $R_{32}^{A}$, $R_{32}$ > $R_{23}^{B}$	$\xi_{32}$ = 0
$R_{33}^{A}$ > 0, $R_{33}^{B}$ > 0, $R_{33}$ > 0	$R_{33}^{A}$ < $R_{33}^{B}$, $R_{33}$ < $R_{33}^{A}$, $R_{33}$ < $R_{33}^{B}$	$\xi_{33}$ < 0
	$R_{33}^{A}$ > $R_{33}^{B}$, $R_{33}$ < $R_{33}^{A}$, $R_{33}$ < $R_{33}^{B}$	$\xi_{33}$ > 0
	$R_{33}^{A}$ = $R_{33}^{B}$, $R_{33}$ < $R_{33}^{A}$, $R_{33}$ < $R_{33}^{B}$	$\xi_{33}$ = 0
$R_{det}^{A}$ > 0, $R_{det}^{B}$ > 0, $R_{det}$ > 0	$R_{det}^{A}$ < $R_{det}^{B}$, $R_{det}$ < $R_{det}^{A}$, $R_{det}$ < $R_{det}^{B}$	$\xi_{det}$ < 0
	$R_{det}^{A}$ > $R_{det}^{B}$, $R_{det}$ < $R_{det}^{A}$, $R_{det}$ < $R_{det}^{B}$	$\xi_{det}$ > 0
	$R_{det}^{A}$ = $R_{det}^{B}$, $R_{det}$ < $R_{det}^{A}$, $R_{det}$ < $R_{det}^{B}$	$\xi_{det}$ = 0

## References

[1] Kondepudi D. (2008). Introduction to Modern Thermodynamics.

[2] Baker R. (2012). Membrane Technology and Application.

[3] Uragami T. (2017). Science and Technology of Separation Membranes.

[4] Vafai K. (2011). Porous Media: Applications in Biological Systems and Biotechnology.

[5] Plawsky J.L. (2020). Transport Phenomena Fundamentals.

[6] Hoogendoorn A., van Kasteren H. (2020). Transportation Biofules: Pathways for Production.

[7] Speight J.G. (2019). Natural Water Remediation: Chemistry and Technology.

[8] Dermirel Y. (2007). Nonequilibrium Thermodynamics: Transport and Rate Processes in Physical, Chemical and Biological Systems.

[9] Nikonenko V.V., Kovalenko A.V., Urtenov M.K., Pismenskaya N.D., Han J., Sistet P., Pourcelly G. (2014). Desalination at Overlimitinng Currents: State-Of-Theart and Perspectives. Desalination.

[10] Kedem O., Katchalsky A. (1958). Thermodynamics Analysis of the Permeability of Biological Membranes to Non-Electrolytes. Biochim. Biophys. Acta.

[11] Katchalsky A., Curran P.F. (1965). Nonequilibrium Thermodynamics in Biophysics.

[12] Kargol A., Kargol M., Przestalski S. (1997). The Kedem-Katchalsky Equations as Applied for Describing Substance Transport Across Biological Membranes. Cell. Mol. Biol. Lett..

[13] Kargol A. (2001). A Mechanistic Model of Transport Processes in Porous Membranes Generated by Osmotic and Hydrostatic Pressure. J. Membr. Sci..

[14] Kargol M., Kargol A. (2003). Mechanistic Formalism for Membrane Transport Generated by Osmotic and Mechanical Pressure. Gen. Physiol. Biophys..

[15] Peusner L. (1986). Studies in Network Thermodynamics.

[16] Elmoazzen H.Y., Elliot J.A.W., McGann L.E. (2009). Osmotic Transport across Cell Membranes in Nondilute Solutions: A New Nondilute Solute Transport Equation. Biophys. J..

[17] Cheng X., Pinsky P.M. (2015). The Balance of Fluid and Osmotic Pressures across Active Biological Membranes with Application to the Corneal Endothelium. PLoS ONE.

[18] Cardoso S.S.S., Cartwright J.H.E. (2014). Dynamic of Osmosis in a Porous Medium. R. Soc. Open Sci..

[19] Kedem O., Caplan S.R. (1965). Degree of Coupling and Its Relation to Efficiency of Energy Conversion. Trans. Faraday Soc..

[20] Caplan S.R. (1965). The Degree of Coupling and Its Relation to Efficiency of Energy Conversion in Multiple-Flow Systems. J. Theor. Biol..

[21] Peusner L. (1983). Hierarchies of Irreversible Energy Conversion Systems: A Network Thermodynamics Approach. I. Linear Steady State without Storage. J. Theor. Biol..

[22] Peusner L. (1985). Hierarchies of Irreversible Energy Conversion Systems II. Network Derivation of Linear Transport Equations. J. Theor. Biol..

[23] Peusner L. (1970). The Principles of Network Thermodynamics: Theory and Biophysical Applications. Ph.D. Thesis.

[24] Oster G., Perelson A., Katchalsky A. (1971). Network Thermodynamics. Nature.

[25] Ślęzak A., Grzegorczyn S., Batko K.M. (2012). Resistance Coefficients of Polymer Membrane with Concentration Polarization. Transp. Porous Media.

[26] Batko K.M., Ślęzak-Prochazka I., Grzegorczyn S., Ślęzak A. (2014). Membrane Transport in Concentration Polarization Conditions: Network Thermodynamics Model Equations. J. Porous Media.

[27] Batko K.M., Ślęzak-Prochazka I., Ślęzak A. (2015). Network Hybrid Form of the Kedem-Katchalsky Equations for Non-Homogenous Binary Non-Electrolyte Solutions: Evaluation of *P_ij_* * Peusner’s Tensor Coefficients. Transp. Porous Media.

[28] Ślęzak-Prochazka I., Batko K.M., Wąsik S., Ślęzak A. (2016). *H** Peusner’s Form of the Kedem-Katchalsky Equations Fon On-Homogeneous Non-Electrolyte Binary Solutions. Transp. Porous Media.

[29] Ślęzak A., Dworecki K., Anderson J.A. (1985). Gravitational Effects on Transmembrane Flux: The Rayleigh-Taylor Convective Instability. J. Membr. Sci..

[30] Ślęzak A., Grzegorczyn S., Jasik-Ślęzak J., Michalska-Małecka K. (2010). Natural Convection as an Asymmetrical Factor of the Transport through Porous Membrane. Transp. Porous Media.

[31] Schlichting H., Gersten K. (2000). Boundary Layers Theory.

[32] Barry P.H., Diamond J.M. (1984). Effects of Unstirred Layers on Membrane Phenomena. Physiol. Rev..

[33] Ślęzak A. (1989). Irreversible Thermodynamic Model Equations of the Transport across a Horizontally Mounted Membrane. Biophys. Chem..

[34] Jasik-Ślęzak J., Olszówka K.M., Ślęzak A. (2011). Estimation of Thickness of Concentration Boundary Layers by oSmotic Volume Flux Determination. Gen. Physiol. Biophys..

[35] Dworecki K., Ślęzak A., Ornal-Wąsik B., Wąsik S. (2005). Effect of Hydrodynamic Instabilities on Solute Transport in Membrane System. J. Membr. Sci..

[36] Ślęzak A., Dworecki K., Jasik-Ślęzak J., Wąsik J. (2004). Method to Determine the Practical Concentration Rayleigh Number in Isothermal Passive Membrane Transport Processes. Desalination.

[37] Ślęzak A., Dworecki K., Ślęzak I.H., Wąsik S. (2005). Permeability Coefficient Model Equations of the Complex: Membrane-Concentration Boundary Layers for Ternary Nonelectrolyte Solutions. J. Membr. Sci..

[38] Dworecki K., Wąsik S., Ślęzak A. (2003). Temporal and Spatial Structure of the Concentration Boundary Layers in Membrane System. Physica A.

[39] Levitt M.D., Strocchi D., Levitt G. (1989). Human Jejunum Unstirred Layer: Evidence for Efficient Luminal Stirring. Am. J. Physiol..

[40] Shibayama T., Morales M., Zhang X., Martínez-Guerrero L.J., Berteloot A., Secomb T.W., Wright S.H. (2015). Unstirred Water Layers and the kinEtics of Organic Cation Transport. Pharm. Res..

[41] Winne D. (1973). Unstirred Layer, Source of Biased Michaelis Constant in Membrane Transport. Biochem. Biophys. Acta.

[42] Batko K., Ślęzak A. (2019). Membrane Transport of Nonelectrolyte Solutions in Concentration Polarization Conditions: H^r^ Form of the Kedem–Katchalsky–Peusner Equations. Int. J. Chem. Eng..

[43] Ślęzak A., Grzegorczyn S., Batko K.M., Bajdur W.M., Makuła-Włodarczyk M. (2020). Applicability of the *L_r_* Form of the Kedem–Katchalsky–Peusner Equations for Membrane Transport in Water Purification Technology. Des. Water Treat..

[44] Lebon G., Jou D., Casas-Vasquez J. (2008). Understanding Non-Equilibrium Thermodynamics. Foundations, Applications, Frontiers.

[45] Batko K.M., Ślęzak A., Bajdur W.M. (2020). The Role of Gravity in the Evolution of the Concentration Field in the Electrochemical Membrane Cell. Entropy.

[46] Baranowski B., Kawczyński A. (1972). Experimental Determination of the Critical Rayleigh Number Inelectrolyte Solutions with Concentration Polarization. Electrochim. Acta.

[47] Baranowski B. (1980). The Electrochemical Analogon of the Benard Instability Studied at Isothermal and Potentiostatic Conditions. J. Non-Equilib. Thermodyn..

